# Competitive Adsorption of Phenolic Acids, Secoiridoids, and Flavonoids in Quercetin Molecularly Imprinted Polymers and Application for Fractionation of Olive Leaf Extracts

**DOI:** 10.1021/acs.jced.3c00543

**Published:** 2024-02-28

**Authors:** Ayssata Almeida, Cláudia Martins, Rolando C. S. Dias, Mário Rui P. F. N. Costa

**Affiliations:** † Centro de Investigação de Montanha (CIMO), Instituto Politécnico de Bragança, Campus de Santa Apolónia, 5300-253 Bragança, Portugal; ‡ LSRE, Faculdade de Engenharia da Universidade do Porto, Rua Roberto Frias s/n, 4200-465 Porto, Portugal

## Abstract

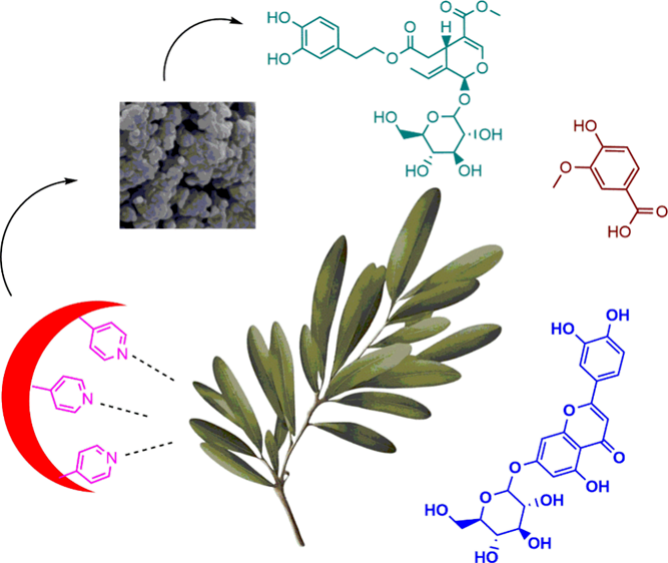

The competitive adsorption of phenolic acids, secoiridoids, and flavonoids in a molecularly imprinted polymer (MIP) functionalized with 4-vinylpiridine (4VP) moieties is analyzed here considering vanillic acid, oleuropein, and quercetin as reference molecules. Measured adsorption isotherms highlight a much stronger binding capacity of the quercetin-MIP particles toward quercetin as compared with vanillic acid and oleuropein. The acquired data were used to design and scale-up sorption/desorption processes aiming at the fractionation of olive leaf extracts. We show that a simple adsorption process, avoiding many pre-preparation steps, is possible when working at a high extract concentration due to the strong binding capacity of the MIP for flavonoids, even when using aqueous mixtures with a large alcoholic content. Solvent-gradient/temperature-swing desorption led to a sequence of fractions with enrichment of non-flavonoids at low alcoholic content while glycosylated flavonoids were enriched in fractions with 40% < alcohol content < 80%. Enrichment factors of 13 and 12 were measured for luteolin-7-*O*-glucoside and apigenin-7-*O*-glucoside, respectively. Flavonoid aglycones were enriched in fractions with alcohol content >80% (enrichment factors >20 for luteolin and quercetin). The findings reported here demonstrate the usefulness of the developed materials and sorption/desorption conditions for agricultural residue valorization and circular bioeconomy.

## 1 Introduction

Around 4.5 million tons of olive leaf are currently generated per year as a byproduct of the olive and olive-oil production. Roughly, these 4.5 million tons of biomass contain 1 million tons of cellulose, 1.5 million tons of lignin, and 1 million tons of bioactive compounds.^1^ While lignin and cellulose fractions find potential important applications for energy generation, materials, and platform chemicals industries, the contained bioactive compounds are worth their valorization for application in the food, feed, chemical, nutraceutical, cosmetic and pharmaceutical sectors.^1^ Secoiridoids (e.g., oleuropein, oleuroside, etc.) and flavonoids (e.g., luteolin, quercetin, and apigenin aglycones as well as the related glycosides), among many other phenolic molecules, besides triterpenoids (erythrodiol, oleanolic acid, etc.), lipids, and volatiles are classes of compounds grounding the interest of olive leaf biomass exploitation for application in the aforementioned industrial fields.

The industries involved in the current efforts for olive leaf valorization are looking for solutions that ensure stable and economically feasible processes. Concerning the bioactive compounds exploitation, besides the variability related with the biomass geographical origin, collection season, and olive leaf variety, methods for efficient extraction and purification should be worked out. A major concern is related with the mixture of diverse compounds that is inevitably obtained apart from the extraction method considered (hydroalcoholic, organic solvents, SCCO_2_, etc.). Therefore, sustainable separation and purification steps should be considered in order to obtain individual molecules or mixtures suitable for practical applications in downstream industries.

Crystallization, membrane filtration, and sorption/desorption processes are methods often considered to concentrate and purify liquid extracts similar to that involved with the valorization of bioactive compounds in olive leaf. The last technique presents some important advantages compared to crystallization (difficult with complex mixtures and energetically highly demanding) and membrane filtration (low selectivity). A lower energy consumption, cyclability, preservation of temperature-sensitive compounds, operational flexibility, amenability to intensification, and industrial scalability make the isolation of target compounds by sorption/desorption processes an attractive possibility for complex extract handling. Nowadays, the combined use of sorption/desorption and membrane (nano)filtration (e.g., for pre-separation of polysaccharides or solvent recovery) is gaining growing room in such kinds of applications.

Actually, cyclic sorption/desorption processes have long been used in industry and analytical applications for concentration, separation, and purification of target compounds. Specifically, its practice with plant extracts and effluents from agricultural related industries, namely, underutilized effluents derived from olive debittering, artichoke washing, and olive oil or natural juices production, among many other examples, is being already observed.^2^ The adsorbent is the core component of such a technical approach, and its selection/design plays a key role for process efficiency and sustainability. High specificity and selectivity toward target compounds, high retention capacities, and ease of regeneration are general requirements often sought. Among different materials that can be used as adsorbents (e.g., activated carbons, silica-based particles, or lignocellulosic/polysaccharides) the synthetic polymer resins associate some key features, namely, high durability, stability, and possibility for their tailoring according to the prospected application. Amberlite, Supellite, and Reillex polymer resins are examples of classes of such materials with huge industrial relevance. The practical use of such kinds of synthetic resins with many systems aiming at plant extracts processing and agricultural effluents valorization and/or treatment (generally wastewater effluents) is already widely reported.^2^

Despite the complexity of the mixture of compounds in the extract to be fractionated and separated, the design of polymeric networks offers the possibility to develop tailored adsorbents with expected higher efficiency. In particular, the composition/functionalization of the resin can be tuned according to the plausible mechanisms of interaction between solutes, solvent(s), and adsorbent, namely, through ionic, hydrophobic (playing a key role with aqueous systems), and hydrogen bonding. Note the important effect of the solvent(s) due to inevitable competition with the solutes for binding with the adsorbent.

In addition to the design of polymer network composition and functionalization, molecular imprinting can also be considered to increase the specificity of the adsorbents toward target molecules. Generically, the molecular imprinting technique aims at the creation of a polymer network with binding cavities stereospecific for selected compounds. For this purpose, a template molecule is present in the reaction media during polymerization for network formation. The removal of the template after polymer formation should leave the sought cavities. The use of a surrogate molecule instead of the target one is a common practice due to potential issues with the participation of the latter in the polymerization reaction (stability, low solubility, very high price, etc.). Due to the potential molecular recognition features of molecularly imprinted polymers (MIPs), their application in a large range of domains has markedly grown in the last decades. Biotechnology, biomedicine, environmental protection, catalysis, and general separation processes are broad examples of areas where MIPs are playing an innovative role.^3−6^ Furthermore, MIPs are also being considered for the valorization of plant extracts and agricultural residues through the isolation of high-added-value compounds (see refs (7−11) and references therein), including also the specific case of olive leaf and olive oil production residues.^12−14^

Herein is reported the development of tailored polymer networks joining functionalization with 4-vinylpyridine (4VP) and molecular imprinting with quercetin as a template. 4VP was considered for functional monomer due to its special binding features that grounds the specific use for the development of advanced polymers in a wide range of applications (wastewater/industrial effluents treatment, enzyme/protein adsorption, electronics, biomedicine, or catalysis^15−22^), including also adsorbents for plant extracts in view of the strong binding with phenolic compounds.^7−12,23^ The rationale for the selection of quercetin is based on its reference structure for many flavonoid molecules and because it is widely available (present in many plants) and is a less expensive molecule, therefore working as a kind of surrogate for flavonoids. Note that, in spite of a possible imperfect selectivity of MIPs and a likely high retention also observed with the correspondent NIPs (non-imprinted polymers) or functional commercial adsorbents, a positive impact of molecular imprinting was demonstrated, not only on specific binding sites formation but also on the improvement of the morphology and textural properties of the adsorbents.^7−12^

In this work, the developed MIP adsorbents were assessed with the competitive adsorption of phenolic acids, secoiridoids, and flavonoids, namely, vanillic acid, oleuropein, and quercetin. The data acquired with these different template molecules were used to design and scale-up sorption/desorption processes considering the MIP particles packed in a preparative column for the enrichment of an industrial olive leaf extract. The efficiency of the developed materials to get high-added-value compounds from olive leaf is demonstrated, namely, through the huge enrichment of aglycone and glycosylated flavonoids.

## 2 Experimental Section

### 2.1 Materials

All chemicals were used as purchased, without further purification. Ethylene glycol dimethacrylate (EGDMA, 98% purity), azobis(isobutyronitrile) (AIBN, 98% purity), and *n*-heptane (>98% purity) were purchased from Sigma-Aldrich. 4-Vinylpyridine (4VP, 95% purity) was provided by Alfa Aesar, and sorbitan mono-oleate (Span 80) was purchased from Panreac. Analytical reagent grade acetonitrile (ACN), dimethylformamide (DMF), acetic acid (AcOH), and methanol (MeOH) were bought from Fisher Scientific, and reagent grade ethanol (EtOH) from PanReac. Quercetin (hydrate, purity 95%) was supplied by Acros Organics. Oleuropein (pure) was purchased from PanReac, and vannilic acid (purity 97%), from Sigma-Aldrich. These standard polyphenols were used in the synthesis and testing of the materials addressed here. The water used in the experiments is ultrapure water supplied by the local laboratory. The industrial olive leaf extract OPA 20% was provided by NATAC (Alcorcón, Madrid, Spain).

### 2.2 Preparation of Quercetin-MIPs through Inverse Suspension Polymerization

A Parr 5100 pressurized glass reactor with a 1 L maximum capacity was used to perform the inverse-suspension polymerization synthesis of the quercetin-MIP particles considered here. This procedure is an extension of our previous works with bulk or precipitation polymerization MIPs preparation.^7−12^ Our goal was scaling up MIP production to the gram-scale in order to work with preparative columns and also control the particle size to prevent potential back-pressure issues when running continuous processes (see sections below). Two separated preparation steps were performed before the reactor charging: (a) In the reaction phase (dispersed phase), quercetin (2.72 g) and 4VP (9.64 mL) were dissolved in ACN/DMF 85/15 (140 mL) and left in an ultrasound bath for 15 min to promote the intermolecular interactions between the quercetin and the 4VP monomer. After, EGDMA (18 mL) and AIBN (1.52 g) were added to the solution for final compounding of the reaction phase. (b) In the continuous phase, *n*-heptane (560 mL) and Span 80 (3.83 g) were mixed and left under vigorous stirring up to the formation of a clear solution. The two solutions were charged to the reactor and degassed during 15 min under stirring (the reactor was equipped with a magnetic drive internal stirrer including double turbine type six-blade impellers at a 45° angle), bubbling a flow of argon through a disperser inside the reactor. The reactor was then pressurized with argon at ∼2 bar, and the polymerization was started by turning on the temperature-control system (set-point = 60 °C). The polymerization was performed with a stirring speed of 400 rpm during a 24 h reaction time. At the end, the solid MIP particles were isolated and purified (cleaning with MeOH/acetic acid in a dialysis bag and Soxhlet extraction with MeOH) with monitoring of the template released by UV–vis spectroscopy and HPLC-DAD. These cleaning steps were performed until the level of the template molecule became too low to be detected. The removal of more than 95% of the initial template is estimated. With polyphenols (here quercetin) the permanent incorporation of the template in the MIPs is plausible at some extent, namely due to the participation in free radical mechanisms.^10^ Purified MIPs were dried (vacuum oven at 45 °C) for polymerization yield assessment, particles characterization (e.g., FTIR), and further use in sorption/desorption runs.

### 2.3 MIP Characterization Using FTIR Spectroscopy

Purified and dried MIP was characterized through Fourier Transform Infrared (FTIR) spectroscopy with a PerkinElmer, model Spectrum Two, instrument. These analyses were directly performed in ATR mode and also with the particles mixed with KBr and pressed into pellets in order to collect the corresponding IR spectra.

### 2.4 SEM Analysis of the MIP Particles

Scanning Electron Microscopy (SEM) characterization of the particles involved in this research was performed at the International Iberian Nanotechnology Laboratory (INL), Braga, using the FIB/SEM system HELIOS Nanolab 450S. SEM imaging was obtained using an electron beam of 3 keV, beam current 25 pA, and field free lens mode.

### 2.5 HPLC-DAD Analysis

An HPLC system (KNAUER) consisting of a gradient pump (P6.1 L) equipped with a degasser, an autosampler (6.1 L), a column thermostat (CT2.1), and a DAD (6.1 L) was used in this research. ClarityChrom was the software allowing control of the HPLC system. The chromatographic analysis was performed using an Ascentis C18 (SUPELCO) column with a particle size of 5 μm and dimensions of 25 cm × 4.6 mm. A gradient of solvents was used as a mobile phase varying from 100% water–ACN (9:1) to 100% water–ACN (1:9) for 45 min. The mobile phase water pH was adjusted to 3 using acetic acid. The flow rate of the chromatographic analyses was 1 mL min^–1^, and the temperature of the column was set at 45 °C.

### 2.6 Packing of the MIP Particles and Sorption/Desorption Runs

MIP particles were packed in two different sized HPLC columns using the slurry method. A small column with dimensions *L* × *D* = 50 mm × 4.6 mm was packed with 290 mg of MIP particles, while a preparative column with dimensions *L* × *D* = 250 mm × 20 mm was packed with 25 g of adsorbent. The small column was used to perform the competitive adsorption studies with the three different standard phenolic compounds considered here, and the fractionation of the industrial olive leaf extract was performed with the packed preparative column. A Knauer HPLC pump (model Azura P 4. 1S, titanium head) with maximum delivery pressure of 40 MPa and flow rate in the range 0.001–10 mL min^–1^ was used to make the flow of the feeding solutions and desorption solvents through the MIP-packed columns (flow rate = 1 mL/min). A column oven was used to define the temperature of the sorption/desorption steps, and the temperature of the feeding solution or desorption solvents was controlled using a thermostatic bath.

### 2.7 Collection, Processing, and Analysis of Samples Resulting from the Fractionation of the Olive Leaf Extracts

The desorption step aimed at fractionation of the industrial olive leaf extract involved the collection of different samples during the solvent-gradient and temperature-swing process. For prescribed running times, samples with specific eluted volume were collected at the column outlet and subsequently processed for recovery of the contained mass of olive leaf extract. The processing of the collected samples included the evaporation of the contained solvent in a rotary evaporator working under a vacuum at 50 °C up to dryness. Afterward, a solution of the recovered solid sample was prepared at a selected concentration (usually 2 mg/mL) for HPLC-DAD analysis and composition determination, including the comparison with the original olive leaf extract and assessment of the fractionation of the phenolic compounds there contained.

### 2.8 Estimation of the Adsorbed Amounts at Equilibrium from Experiments with MIP Competitive Adsorption of Vanillic Acid, Oleuropein, and Quercetin

The estimation of the adsorbed amounts of vanillic acid, oleuropein, and quercetin at equilibrium from experimental data concerning the MIP retention was performed using four different methods. (i) HPLC-DAD analysis of the global liquid solution after percolation through the column and calculation of the non-retained amount for each compound. The adsorbed amount was obtained by difference with respect to the initial solution. (ii) HPLC-DAD analysis of the global liquid solution resulting from the joining of all desorbed fractions after column saturation. The adsorbed amount for each compound is directly obtained. (iii) Numerical integration of the experimental adsorption breakthrough curves for the upload process dynamics (the makima and trapz functions of MATLAB were used with these calculations). (iv) Numerical integration of the experimental curves for the desorption process dynamics (the makima and trapz functions of MATLAB were also used with these calculations). Average adsorbed values were taken with these four measurements, and deviations below 8% were observed in the different experimentalruns. Below, in the Results and Discussion, further details on the experimental procedure adopted in these adsorption studies, as well as the accuracy of the equilibrium measurements, are given. The assessment of the repeatability of the adsorption measurements with standard polyphenols was not performed, because the reproducibility of the sorption/desorption process with the MIP particles was evaluated in the context of the processing of olive leaf extracts, as discussed below in Section 3.3.

## 3 Results and Discussion

### 3.1 Materials Characterization

**Figure 1:**
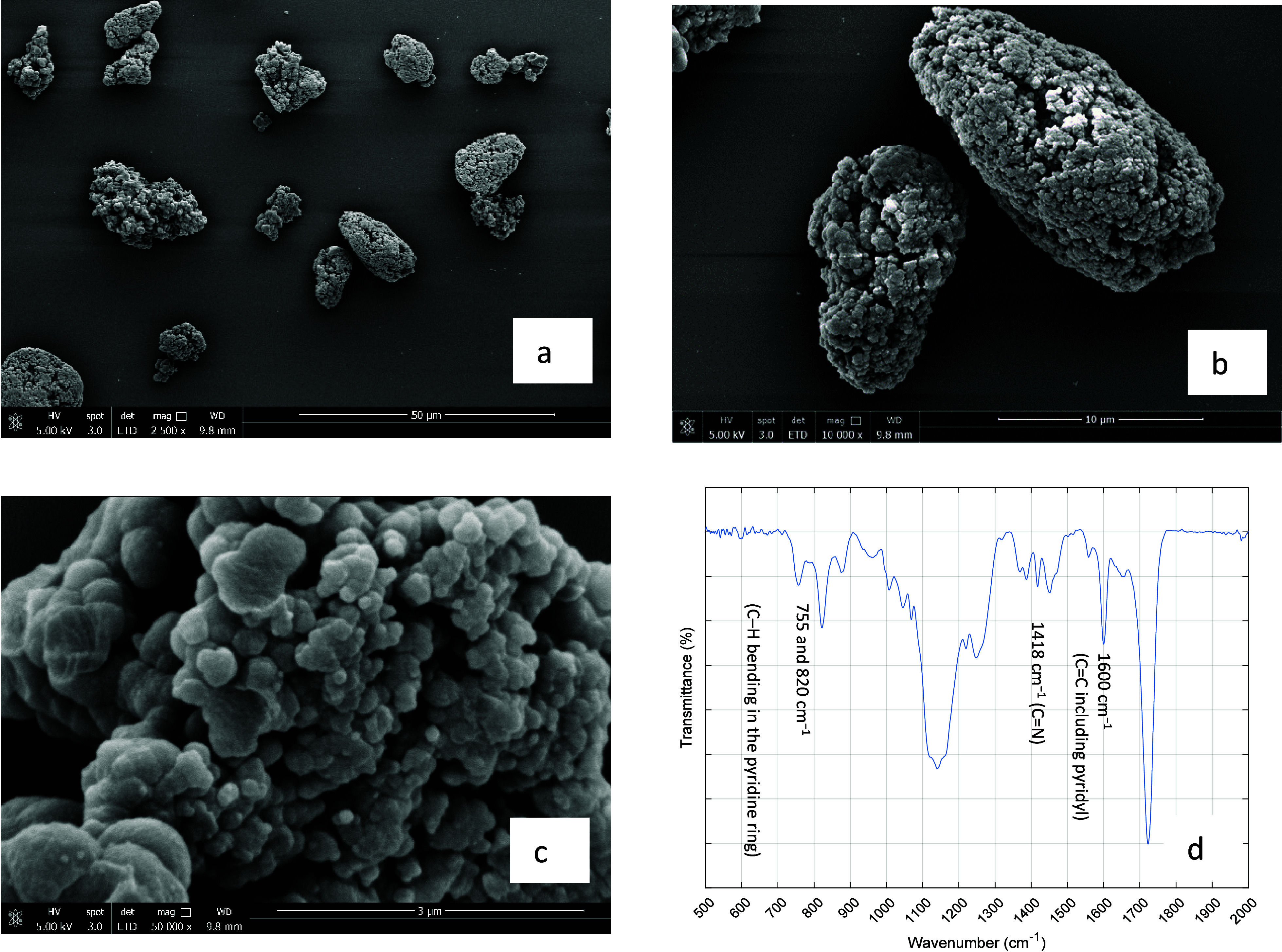
SEM images of the MIP particles produced through inverse-suspension polymerization (a–c) and FTIR analysis of these polymer networks (d).

In Figure 1 are presented SEM images of the MIP particles produced through inverse-suspension polymerization (panels a–c) and also the FTIR analysis of these polymer networks (panel d). The SEM images show the formation of particles with size higher than 10 μm (Figure 1(a),(b)) that result from the agglomeration of smaller nucleus with size <1 μm (Figure 1(c)) born and grown inside each droplet of the dispersed polymerization phase. The formation of individual particles with size <1 μm is often observed when using precipitation polymerization,^7−11^ the application of which in continuous sorption/desorption processes is potentially hindered due to back-pressure issues. Therefore, here was explored the formation of higher size particles through inverse suspension morphology which allows the running of high-pressure sorption/desorption processes. At the same time, the extant smaller nucleus in these larger particles allows preservation of a high surface area for mass transfer and the enhanced accessibility of potential molecularly imprinted sites. The selection of the polymerization technique depends also on the common solubility of all reactants including the template (usually a major problem), and the polymerization mechanism adopted here allows the running with ACN/DMF in the dispersed phase. This synthesis approach looks for balance on these issues (the grinding of the MIPs associated with bulk polymerization is also avoided) and offers a practical method for the large-scale production of the imprinted particles with a morphology suitable for application in continuous sorption/desorption processes, as demonstrated below.

The FTIR analysis of the MIP particles presented in Figure 1(d) demonstrates the incorporation in the polymer networks of the moieties of the functional monomer 4VP and also of the cross-linker EGDMA. FTIR vibrational assignments at 755 and 820 cm^–1^, both corresponding to the C–H bending in the pyridine ring, are indicators of the successful incorporation of 4VP moieties in the synthesized materials. Moreover, the characteristic peak at 1418 cm^–1^ identifies the C=N group and, undoubtedly, the presence in the polymer of the pyridine heterocyclic ring that plays a critical role in the adsorption performance of the materials designed and synthesized. Note that the assignment at 1600 cm^–1^ includes not only the vibrational assignment of pyridyl C=C but also the potentially unreacted C=C double bonds of moieties of EGDMA in the MIPs. Characteristic peaks of the latter appear clearly at 1720 and 1130 cm^–1^, which are assigned to the functional groups C=O and C–O, respectively, confirming the polymerization of the cross-linker and network formation.

### 3.2 MIP Competitive Adsorption of Vanillic Acid, Oleuropein, and Quercetin

Table 1 summarizes the set of experiments performed to acquire data on the competitive adsorption of vanillic acid, oleuropein, and quercetin in the developed MIP particles. These three different template molecules were selected because they are representative examples of compounds belonging to the classes of phenolic acids, secoiridoids, and flavonoids, respectively, that are commonly found in olive leaf extracts. Two hydroalcoholic compositions, namely, EtOH/Water 50/50 and 80/20 (v/v), were considered for sorption because the use of these solvents is “Generally Recognized As Safe (GRAS)”, especially in purification processes for non-toxic applications such as food, cosmetics, or pharmaceuticals. With EtOH/Water 50/50, the upper range for the solutes concentration (0.5 mM) approaches the limit for quercetin solubility in this mixture. With EtOH/Water 80/20, the concentration of solutes could be extended to 2 mM. In such a solvent, a concentration as high as 10 mg/mL is possible for solubilization of an olive leaf extract with 20% oleuropein (a typical industrial extract), which corresponds to a concentration of oleuropein ∼3.7 mM. Therefore, at 2 mM, a range for processing of an olive leaf extract at high concentration was approached, preserving at the same time a similar solubility for quercetin. Note that with the analysis of the competitive adsorption of the three molecules our option was for the use of equimolar feeding solutions. The dissimilar concentration of the competitive compounds is later analyzed with the processing of an olive leaf extract.

**Table 1:** Description of the Set of Experiments Performed to Analyze the Competitive Adsorption of Vanillic Acid, Oleuropein, and Quercetin in the Developed MIP Particles

	Sorption					Desorption	
Run	*T* (°C)	Solvent	Quercetin (mM)	Vanillic Acid (mM)	Oleuropein (mM)	*T* (°C)	Solvent
1	25	EtOH/Water 50/50	0.125	0.125	0.125	25	EtOH/Water 50/50 + MeOH/Acetic acid 90/10
2			0.25	0.25	0.25		
3			0.5	0.5	0.5		
4		EtOH/Water 80/20	0.2	0.2	0.2		EtOH/Water 80/20 + MeOH/Acetic acid 90/10
5			1	1	1		
6			2	2	2		
7	45		0.2	0.2	0.2	45	
8			1	1	1		
9			2	2	2		

Two temperatures were considered in the sorption–desorption studies, specifically 25 and 45 °C, as detailed in Table 1. The use of relatively low temperatures is important to prevent bioactive compound degradation and also to decrease energetic costs associated with such industrial processing. In the desorption step, after the use of the same solvent of the loading (EtOH/Water 50/50 or 80/20), a stronger finishing elution stage with MeOH/Acetic acid 90/10 (v/v) was considered to achieve the total cleaning of the adsorbent, as detailed below.

**Figure 2:**
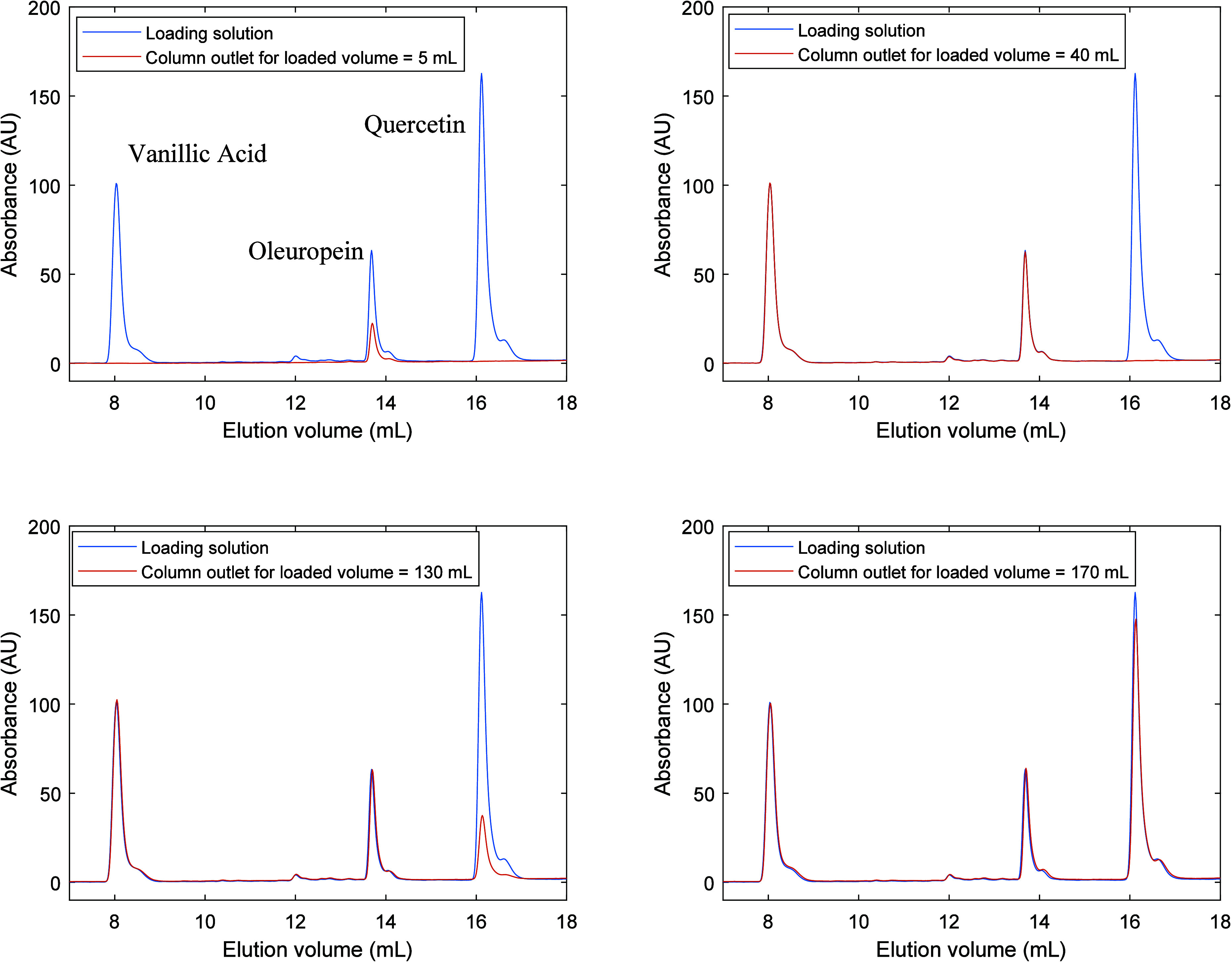
HPLC analysis for samples collected at the MIP column outlet during the loading of a solution containing vanillic acid, oleuropein, and quercetin according to conditions of Run 1.

**Figure 3:**
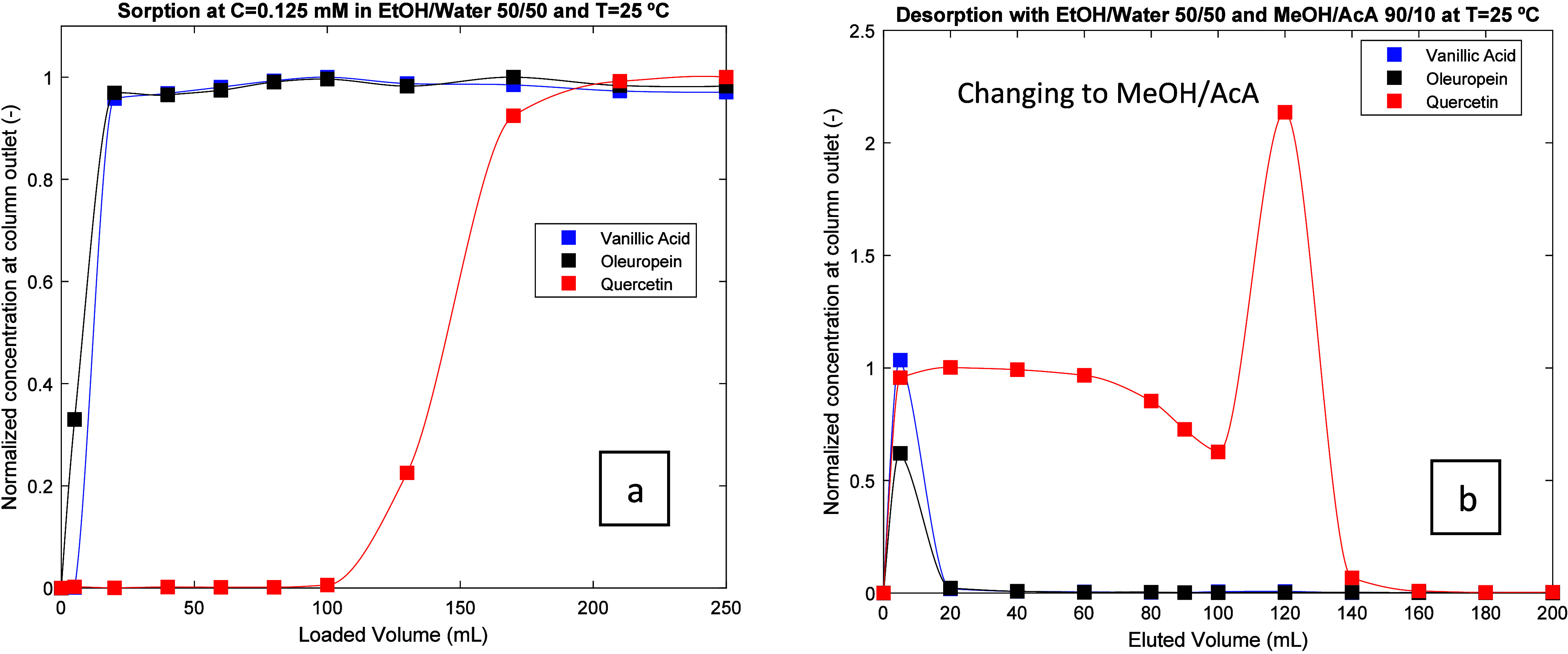
(a) Experimental breakthrough curve for the competitive adsorption of vanillic acid, oleuropein, and quercetin in the MIP adsorbent according to conditions of Run 1. (b) Experimental desorption profile obtained for the same experiment. Lines in the plots were included to guide the eye.

Figure 2 illustrates with a specific example (Run 1 in Table 1) the measurement approach considered here for the analysis of the competitive adsorption of vanillic acid, oleuropein, and quercetin in the MIP particles. During the column saturation process, samples were collected at the column outlet for prescribed loaded volumes and analyzed through HPLC-DAD for quantification of the contained compounds. The example in Figure 2 clearly shows an early saturation of the material with oleuropein, after with vanillic acid, while a strong retention of quercetin in the adsorbent is observed (only after the loading of 170 mL does the saturation for quercetin start to be approached). With this analytical information, data for the experimental breakthrough curve concerning the competitive adsorption of vanillic acid, oleuropein, and quercetin in the MIP adsorbent were acquired, as illustrated in Figure 3(a), also taken Run 1 as example. The breakthrough curve shows a markedly high retention on quercetin in the developed material, as compared with oleuropein and vanillic acid.

Similar data for the competitive breakthrough curves corresponding to all the experiments described in Table 1 were obtained and are presented in the Supporting Information (Figures S1–S9).

**Table 2:** Adsorption Equilibrium Measurements for MIP Competitive Adsorption of Vanillic Acid, Oleuropein, and Quercetin in the Developed MIP Particles

	Running conditions			Adsorbed amounts (μmol/g) and corresponding uncertainties					
Run	Liquid concentration (mM)	*T* (°C)	Solvent	Quercetin		Vanillic Acid		Oleuropein	
1	0.125	25	EtOH/Water 50/50	61.2	1.8	7.0	0.4	5.0	0.4
2	0.25			91.4	3.7	15.0	0.9	8.4	0.4
3	0.5			154.1	3.1	28.1	1.7	22.9	1.6
4	0.2		EtOH/Water 80/20	19.1	1.0	3.9	0.2	2.3	0.1
5	1			72.6	2.5	23.9	1.1	18.2	1.4
6	2			120.9	4.8	39.6	2.4	28.6	1.9
7	0.2	45		15.0	0.9	3.5	0.2	3.5	0.2
8	1			58.9	2.2	17.9	1.1	15.4	1.2
9	2			100.5	4.1	33.4	2.2	29.8	2.1

An equivalent measurement approach was adopted for the desorption (MIP column previously saturated) by collecting at the column outlet samples at prescribed elution volumes and subsequent compositional analysis by HPLC-DAD (see an illustration in Figure S10 of the Supporting Information document). With this information, experimental desorption profiles for all experiments were also acquired, as presented in Figure 3(b) for Run 1 (Figures S1–S9 in the Supporting Information contain the desorption profiles for all experiments). These results also clearly show the strong binding of quercetin in the MIPs as compared with oleuropein (the weakly bind molecule) and vanillic acid. Note that all the desorption runs included a late desorption step considering elution with MeOH/Acetic acid 90/10, as ascribed in plot 3(b) and similar plots in the Supporting Information file. When changing the desorption to MeOH/Acetic acid, a boost in quercetin release is observed (oleuropein and vanillic acid are eluted at the early stages), and if needed, this approach can be considered for the speed-up of the elution of the MIP strongly retained compounds (see the section below concerning the processing of an olive leaf extract). The mass balance on the loaded and eluted amounts of each compound in the nine experiments was used to assess the efficiency of mass recovery and the precision of the saturation values for the different running conditions. Agreement >92% between the loaded and eluted quantities was observed for all the experiments.

**Figure 4:**
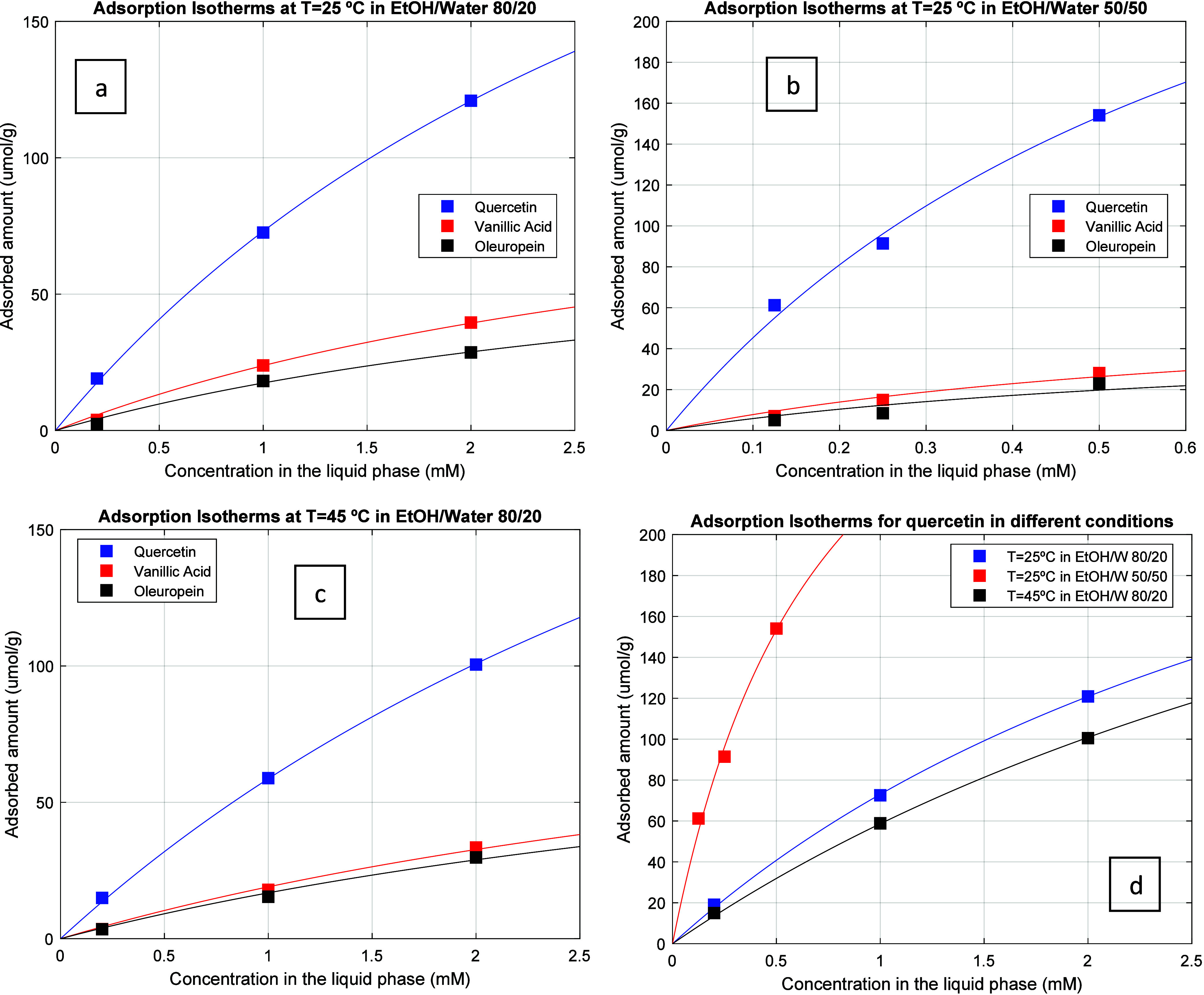
Experimental adsorption isotherms measured for the competitive adsorption of vanillic acid, oleuropein, and quercetin in the MIP adsorbent in different conditions. (a) *T* = 25 °C and EtOH/Water 80/20 as solvent. (b) *T* = 25 °C and EtOH/Water 50/50 as solvent. (c) *T* = 45 °C and EtOH/Water 80/20 as solvent. (d) Comparison of the adsorption isotherms for quercetin in the three different running conditions considered. The lines in the plots correspond to the fitting of experimental data with the extended Langmuir model eq 1 and modified version eqs 2–4.

The assembly of experimental data collected up to column saturation was used to calculate the amount of each compound adsorbed at equilibrium and therefore to get insights on the adsorption isotherms, as presented in Figure 4(a–c) for the different running conditions considered, namely, (a) *T* = 25 °C and EtOH/Water 80/20, (b) *T* = 25 °C and EtOH/Water 50/50, and (c) *T* = 45 °C and EtOH/Water 80/20. In Figure 4(d) is presented the comparison of the adsorption isotherms for quercetin in these three different running conditions. These isotherms, similar to the previously discussed breakthrough curves and desorption profiles, show a much higher retention of quercetin as compared with vanillic acid and oleuropein (the less retained compound). It is also worth mentioning the observed effect of hydrophobic interactions on retention increase (comparison of EtOH/Water 80/20 and EtOH/Water 50/50 at *T* = 25 °C) and, as expected, the lower adsorption at *T* = 45 comparatively to *T* = 25 °C. Note, however, the lower range of concentration possible with EtOH/Water 50/50 in order to fulfill total solubility, as discussed above.

In Table 2 are presented the adsorption equilibrium measurements performed with the MIP competitive adsorption of vanillic acid, oleuropein, and quercetin (left column for each compound in μmol/g) with the developed MIP particles. Estimated uncertainties, according to the methods described in Section 2.8 (see Supporting Information for a calculation example), are also reported in the right column for each compound.

Additional insights on the adsorption isotherms were worked out through the data fitting to an extended Langmuir model for competitive adsorption:^24^
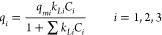
1Indices *i* = 1, 2, 3 above correspond to quercetin, vanillic acid, and oleuropein, respectively.

On the other hand, the *q*_*mi*_ represent the maximum adsorption capacity for each compound and *k*_*Li*_ the affinity constants (ratio of the sorption and desorption rates) that correlate with the bonding energy characteristic for each molecule.

In Table 3 are presented the numerical values for the parameters obtained through the fitting of the competitive equilibrium adsorption experimental data according to the extended Langmuir model. These parameters describe numerically the differences in the adsorption of the three compounds mentioned above, specifically the much high binding strength of quercetin comparatively to oleuropein and vanillic acid. Note, however, that the experimental data for the isotherms are in a region of low concentration due to the aforementioned solubility restrictions. Therefore, the values for *q*_*m*_ are estimations based on the experimental data just for the initial linear region of the isotherms. The impact of temperature and water content in solvent (causing hydrophobic effects) on adsorption becomes clear when the different *k*_*L*_ values are compared (see the high value for quercetin in EtOH/W 50/50 at *T* = 25 °C). Especially meaningful in this context are the values for *q*_*m*_*k*_*L*_ (slope of the isotherm in the linear region) as observed in Table 3. The order for adsorption quercetin > vanillic acid > oleuropein, in EtOH/W 50/50 > EtOH/W 80/20 and at *T* = 25 > *T* = 45 °C is well represented by the comparison of the *q*_*m*_*k*_*L*_ for the different isotherms.

**Table 3:** Fitting Parameters for the Competitive Adsorption of Quercetin, Vanillic Acid, and Oleuropein in the Developed MIP Particles, According to an Extended Langmuir Model (Eq 1)^a^

		Quercetin			Vanillic Acid			Oleuropein		
*T*	Solvent	*q*_*m*_	*k*_*L*_	*q*_*m*_*k*_*L*_	*q*_*m*_	*k*_*L*_	*q*_*m*_*k*_*L*_	*q*_*m*_	*k*_*L*_	*q*_*m*_*k*_*L*_
25	EtOH/W 80/20	666	0.139	92.3	439	0.068	29.9	387	0.057	22.0
25	EtOH/W 50/50	611	0.843	514.8	319	0.277	88.4	279	0.237	66.2
45	EtOH/W 80/20	700	0.100	70.0	465	0.049	22.8	441	0.046	20.1

a The temperature *T* is expressed in °C, *q*_*m*_ in μmol/g, and *k*_*L*_ in mM^–1^.

The above analyzed extended Langmuir model uses several simplifying assumptions, namely, a homogeneous surface, no interaction between adsorbed species, and equal availability of adsorption sites to all species. However, according to Jain and Snoeyink,^25^ this theory is not really accounting for competition and new terms were added to the original equations (eq 1 above) to describe the distinction between the competitive/non-competitive adsorbed amounts. This possibility was also assessed here considering a new adsorption model, derived from the Jain and Snoeyink approach,^25^ that is described by eqs 2–4 below. The first term in eq 2 is used to describe the non-competitive adsorption of quercetin. This hypothesis was considered due to the potential specific imprinted cavities for quercetin in the polymer network. The second term in eq 2 describes the amount of quercetin adsorbed in competition with vanillic acid and oleuropein. On the other hand, eqs 3 and 4 represent the adsorbed amounts of vanillic acid and oleuropein, respectively, that is considered to occur in competition with quercetin for both cases.
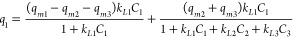
2
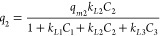
3
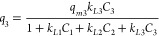
4

As before, the experimental data for the isotherms (Figure 4) were fitted to the model represented by eqs 2–4 considering the constraint *q*_*m*1_ – *q*_*m*2_ – *q*_*m*3_ > 0. A very good fitting quality was again achieved (see lines in Figure 4), and in fact, only residual differences in the parameter numerical values are observed in comparison with the extended Langmuir model (eq 1), as can be concluded through a comparison of Table 3 and Table 4 (rounded numerical values are presented). These calculations indicate that the adsorbed amount of quercetin without competition, proportional to *q*_*m*1_ – *q*_*m*2_ – *q*_*m*3_ in eq 2, is much smaller than the competitive one (proportional to *q*_*m2*_ + *q*_*m3*_). These results suggest that the imprinted cavities are also available for the competitive sorption of vanillic acid and oleuropein.

**Table 4:** Fitting Parameters for the Competitive Adsorption of Quercetin, Vanillic Acid, and Oleuropein in the Developed MIP Particles, According to a Modified Extended Langmuir Model (Eqs 2–4)^a^

		Quercetin			Vanillic Acid			Oleuropein		
*T*	Solvent	*q*_*m*_	*k*_*L*_	*q*_*m*_*k*_*L*_	*q*_*m*_	*k*_*L*_	*q*_*m*_*k*_*L*_	*q*_*m*_	*k*_*L*_	*q*_*m*_*k*_*L*_
25	EtOH/W 80/20	742	0.124	92.3	389	0.077	30.1	353	0.062	22.0
25	EtOH/W 50/50	606	0.849	514.8	325	0.272	88.3	281	0.235	66.2
45	EtOH/W 80/20	800	0.088	70.0	406	0.056	22.7	394	0.051	20.1

a The temperature *T* is expressed in °C, *q*_*m*_ in μmol/g, and *k*_*L*_ in mM^–1^.

**Figure 5:**
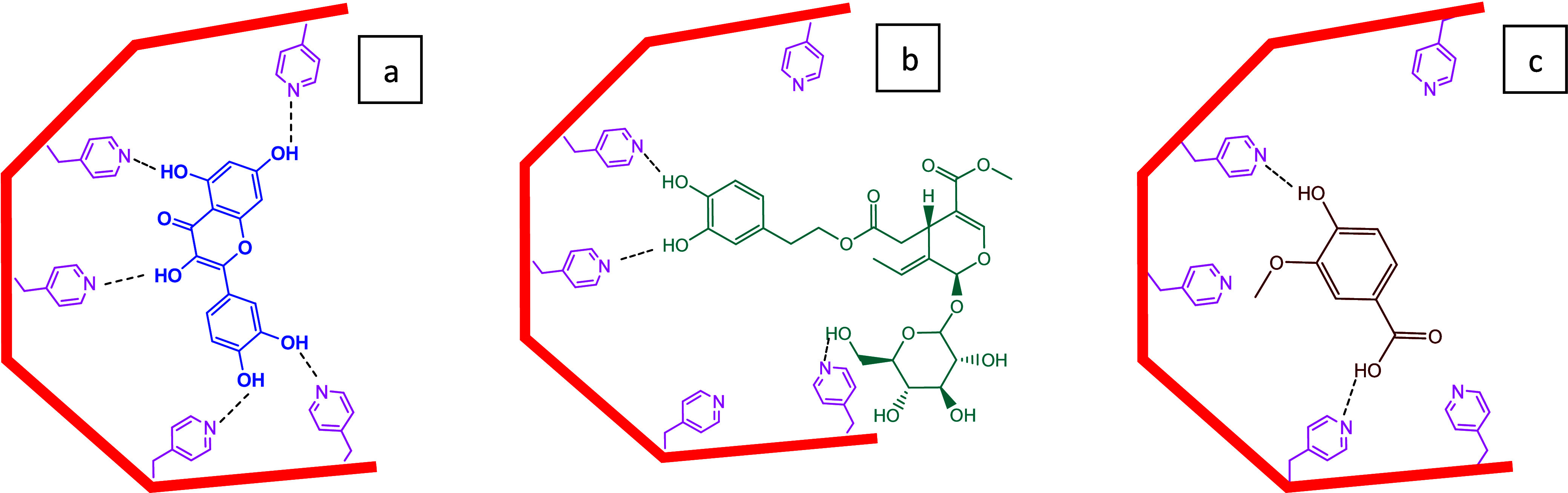
Simple sketch for some plausible intermolecular interactions of (a) quercetin, (b) oleuropein, and (c) vanillic acid with the 4VP-rich molecularly imprinted polymer network synthesized in this work. A multivalent hydrogen bonding of the phenolic groups in quercetin with the pyridyl groups in the polymer network is possible. A lower number of pyridyl–phenolic hydrogen bonding interactions are expected for oleuropein and vanillic acid, and steric hindrance is possible for oleuropein. Hydrophobic effects, competitive interaction of the polymer network functional groups with solvent molecules, are other factors that impact the solute adsorption process. In spite of the expected effect of the stereospecificity of the imprinted cavities in the MIP polymer network, similar interactions are possible with non-imprinted adsorbents of the same kind (NIPs).

In Figure 5 is presented a simple sketch for the interactions of the three standard molecules with the 4VP-rich polymer network helping with the rationale grounding their different adsorption. A multivalent hydrogen bonding of the phenolic groups in quercetin with the pyridyl groups in the polymer network is possible, but a lower number of pyridyl–phenolic hydrogen bonding interactions are expected for oleuropein and vanillic acid. Additionally, phenolic groups have an increased acidity as compared to hydroxyl groups in the sugar moieties (see oleuropein molecular structure in Figure 5). Indeed, with ionization of phenolic hydroxyls, the negative charge and a set of the lone pair electrons in the phenoxide oxygen are delocalized by resonance to the carbons on the aromatic ring. Therefore, a much higher interaction of the nucleophilic pyridyl group (weak base) is expected with the phenolic hydroxyls compared to those in the sugar rings of oleuropein. Due to its larger size, steric hindrance is possible for the interaction of oleuropein with the polymer network; namely, specific cavities formed through molecular imprinting. For vanillic acid, in spite of the low number of phenolic hydroxyls, the higher acidity of the carboxylic group should contribute to increasing the interaction with the weakly basic pyridyl moieties in the polymer network. Besides these kinds of interactions, hydrophobic effects (see comparison EtOH/W 50/50 with EtOH/W 80/20) and competitive interaction of the polymer network functional groups with solvent molecules are other factors with an impact on the solutes’ adsorption process. It should also be mentioned that, in spite of the particular expected effect of the stereospecificity of the imprinted cavities in the MIP polymer network, similar interactions are possible with non-imprinted adsorbents of the same functional kind (non-imprinted polymers (NIPs) based on 4VP). As for our knowledge, the measurement of the binding strength of 4VP with quercetin is not available (namely, in the context of the reaction conditions used here for MIPs formation), and the same applies to the binding strength between the pyridyl moieties in the polymer network and the polyphenols considered in this work. Previous studies addressed the binding between pyridine and phenol, reporting the observations of hydrogen bonding and the hydrophobic association. More recently, using DFT calculations, the energy of the adduct of electron and phenol–pyridine complex was estimated, suggesting it is located mainly in the pyridine ring.^41^

In Table 5 are presented data available in the literature for the adsorption equilibrium of standard phenolic compounds found in olive leaf in view of a comparison with the outcomes of the present work. Data presented in Table 5 show the important impact of the working conditions, namely, the kind of adsorbent and solvent, in the adsorption of phenolic compounds belonging to the different classes that are typically observed in olive leaf. Note the good capability for adsorption of these compounds by 4VP based industrial adsorbents, even in hydroalcoholic mixtures with high alcohol content (see, e.g., the comparison between Reillex and DAX8 resins when working with EtOH/W 80/20). The data in Table 5 also demonstrate the benefits of molecular imprinting at the top of the consideration of 4VP polymer networks. Very good results for oleuropein retention and application of a temperature-swing purification method with a MIP based on styrene, ethylene glycol dimethacrylate, and 1-(4-vinylphenyl)-3-(3,5-bis(trifluoromethyl)phenyl)urea (BTPU), prepared through suspension polymerization, were obtained by Didaskalou et all.^13,14^ The use of other functional monomers, such as methacrylic acid, acrylamide, methacryloyl benzotriazole-Cu(ii) metal-chelate, and *N*-methacryloyl-(l)-histidine methylester-Cu(ii) metal-chelate was also scrutinized with oleuropein as the imprinting template^13^ while the pair BTPU/styrene was also used with imprints for the luteolin and pinoresinol templates.^14^ Using ethyl acetate as solvent, retention capabilities of 168.1, 302.5, and 186.2 μmol/g were measured for oleuropein, luteolin, and pinoresinol, respectively, at *C*_*e*_ = 0.5 mM (see Table 5 and refs (13) and (14) for full information on the isotherms). Note that, in other related studies with application of MIPs for the removal of contaminants in edible vegetable oils, an array of additional functional monomers (e.g., itaconic acid, (3-mercaptopropyl)trimethoxysilane, etc.) was considered to get molecular recognition toward the target compounds.^38,39^

**Table 5:** Equilibrium Adsorbed Amounts of Standard Compounds in Different Adsorbents and Working Conditions in Comparison with the Values Reported in This Work (*T* = 25 °C for All Systems)

Compound	Adsorbent	Solvent	Method	*C*_*e*_ (mM)	*q*_*e*_ (μmol/g)	ref
Quercetin	MIP-QUER	EtOH/W 50/50	Competitive in packed column	0.5	154.1	This work
Oleuropein	MIP-QUER	EtOH/W 50/50	Competitive in packed column	0.5	28.1	This work
Vanillic Acid	MIP-QUER	EtOH/W 50/50	Competitive in packed column	0.5	22.9	This work
Quercetin	MIP-QUER	MeOH/W 50/50	Individual in packed column	0.07	109.6	(9)
Quercetin	DAX8	MeOH/W 50/50	Individual in packed column	0.07	13.8	(9)
Quercetin	DAX8	EtOH/W 80/20	Individual in batch mode	0.17	0.7	(11)
Quercetin	Reillex 425	EtOH/W 80/20	Individual in batch mode	0.08	2.5	(11)
Quercetin	Reillex 402	EtOH/W 80/20	Individual in batch mode	0.03	3.4	(11)
Quercetin	MIP-QUER	EtOH/W 80/20	Individual in batch mode	0.02	3.6	(11)
Oleuropein	MIP-OPA	Ethyl Acetate	Individual in batch mode	0.5	168.1	(13,14)
Luteolin	MIP-LUT	Ethyl Acetate	Individual in batch mode	0.5	302.5	(14)
Pinoresinol	MIP-PIN	Ethyl Acetate	Individual in batch mode	0.5	186.2	(14)

Here we are adopting the use of 4VP MIPs with hydroalcoholic mixtures as solvents, particularly ethanol/water mixtures, due to the industrial practice for the production of olive leaf extracts as well as the more straightforward application of the fractionated products in feed, food, or cosmetic industries. The good performance of the MIPs developed here to work under such conditions is explored below with the fractionation of real olive leaf extracts.

### 3.3 Application for Fractionation of Olive Leaf Extracts

The analysis presented above for the sorption/desorption of the standard molecules vanillic acid, oleuropein, and quercetin in the developed MIP particles was exploited for the fractionation of olive leaf extracts with complex composition. In particular, the huge difference observed with the adsorption/desorption profile of quercetin as compared with the other two molecules was attempted for the enrichment of flavonoids in olive leaf. As a matter of fact, regardless of the extraction technique considered (e.g., maceration, Soxhlet, ultrasound-assisted with hydro-alcoholic mixtures or organic solvents, supercritical CO_2_ extraction, etc.), a complex mixture of compounds belonging to different classes is always obtained at the end.^26−37^ This is a consequence of the plethora of different classes of chemicals found in the olive leaf, for which total typical composition includes lignin (35%), polyphenols (20%), cellulose and fermentable sugars (18%), proteins and minerals (9%), hemicellulose (8%), triterpenic compounds (5%), and a non-polar fraction (essential oil, lipids, and chlorophylls) at a total of 5%. Whereas lignin, cellulose/hemicellulose, and fermentable sugars find important applications for energy industries, materials, platform chemicals, etc., the valorization of this biomass in the framework of circular bioeconomy is also being attempted through the refining of products with application in food, feed, pharmaceuticals, and cosmetics. This valorization route explores as target compounds the olive leaf contained phenolic molecules, triterpenes, triterpenoids, and triterpenic acids (erythrodiol, oleanolic acid, etc.) besides fatty acids, alkanes, and essential oils (e.g., non-terpenic alcohols, non-terpenic aldehydes, sesquiterpene hydrocarbons, oxygen-containing monoterpenes, etc).

**Figure 6:**
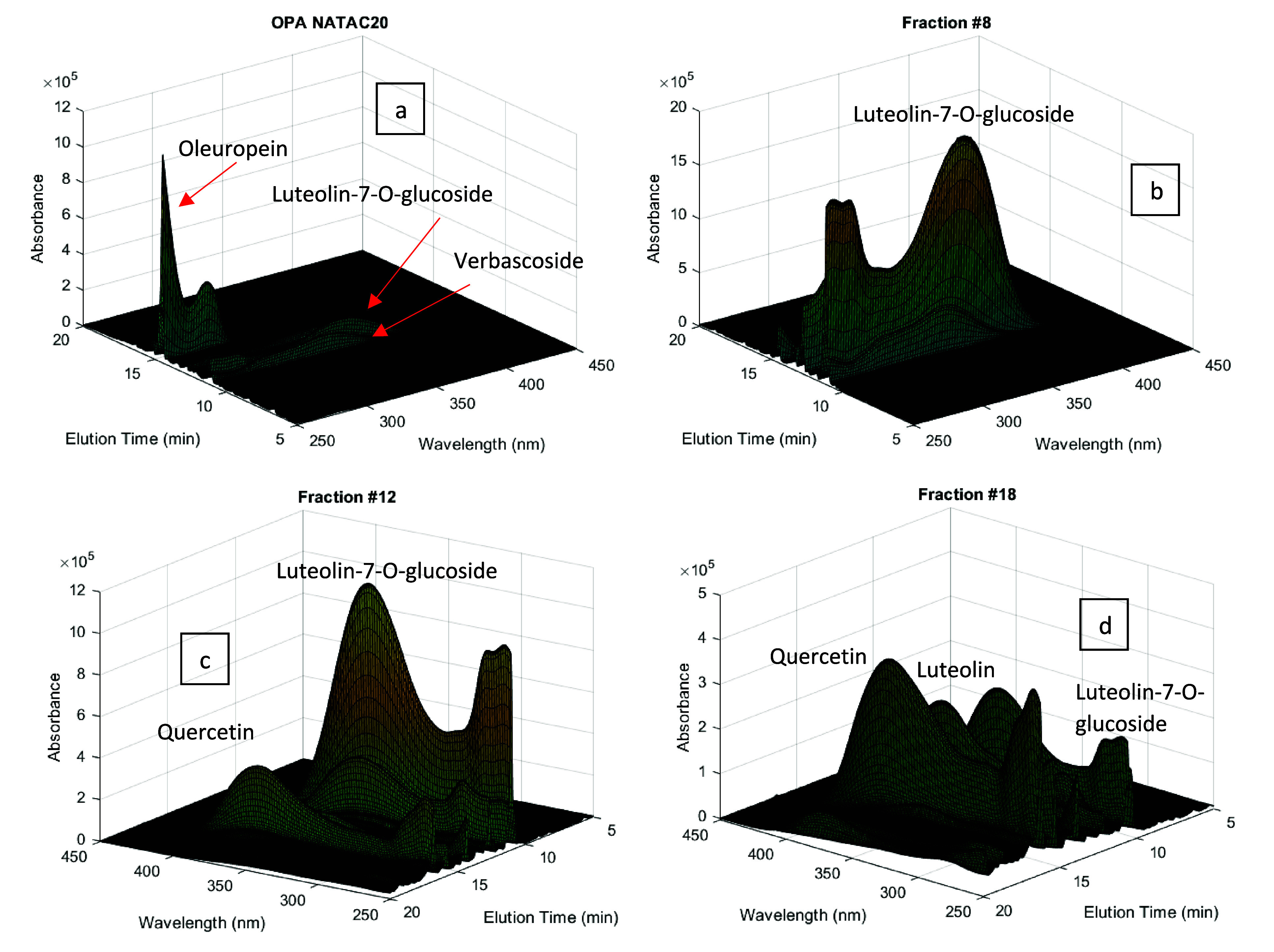
HPLC-DAD analysis for industrial OPA NATAC 20% olive leaf extract (a). Fractions produced through the sorption/desorption processing of the OPA NATAC 20% extract with the developed MIP particles: (b) fraction 8; (c) fraction 12; (d) fraction 18. (See also Figure 8.)

Hydroalcoholic extracts of olive leaf are particularly rich in phenolic compounds including simple phenols and phenolic acids (e.g., tyrosol, hydroxytyrosol, gallic acid, and vanillic acid), secoiridoids (e.g., oleuropein), lignans (e.g., pinoresinol), and flavonoids (e.g., luteolin glucosides, luteolin, quercetin, and apigenin). This is also the reference composition for the industrial olive leaf extract used here (NATAC OPA 20%), for which HPLC-DAD analysis is presented in Figure 6(a). Oleuropein is the predominant phenolic compound in this extract (∼20 wt %) while luteolin-7-*O*-glucoside and verbascoside are also directly identified in the chromatogram presented in Figure 6(a). However, an enlarged view of the HPLC-DAD analysis allows us to discern plenty other different minority compounds such as oleuroside, luteolin-4-*O*-glucoside, diosmetin-4-*O*-glucoside, apigenin-7-*O*-glucoside, quercetin, and luteolin, among many others.^12^

**Figure 7:**
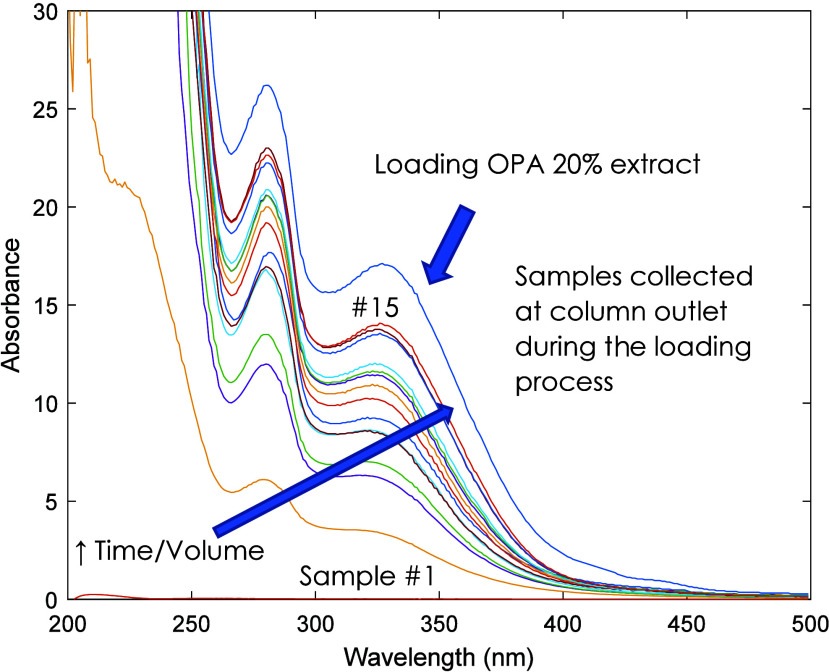
UV–vis monitoring of the loading of the OPA NATAC 20% extract in EtOH/water at 5 mg/mL performed using the MIP-packed preparative column at 25 °C.

**Figure 8:**
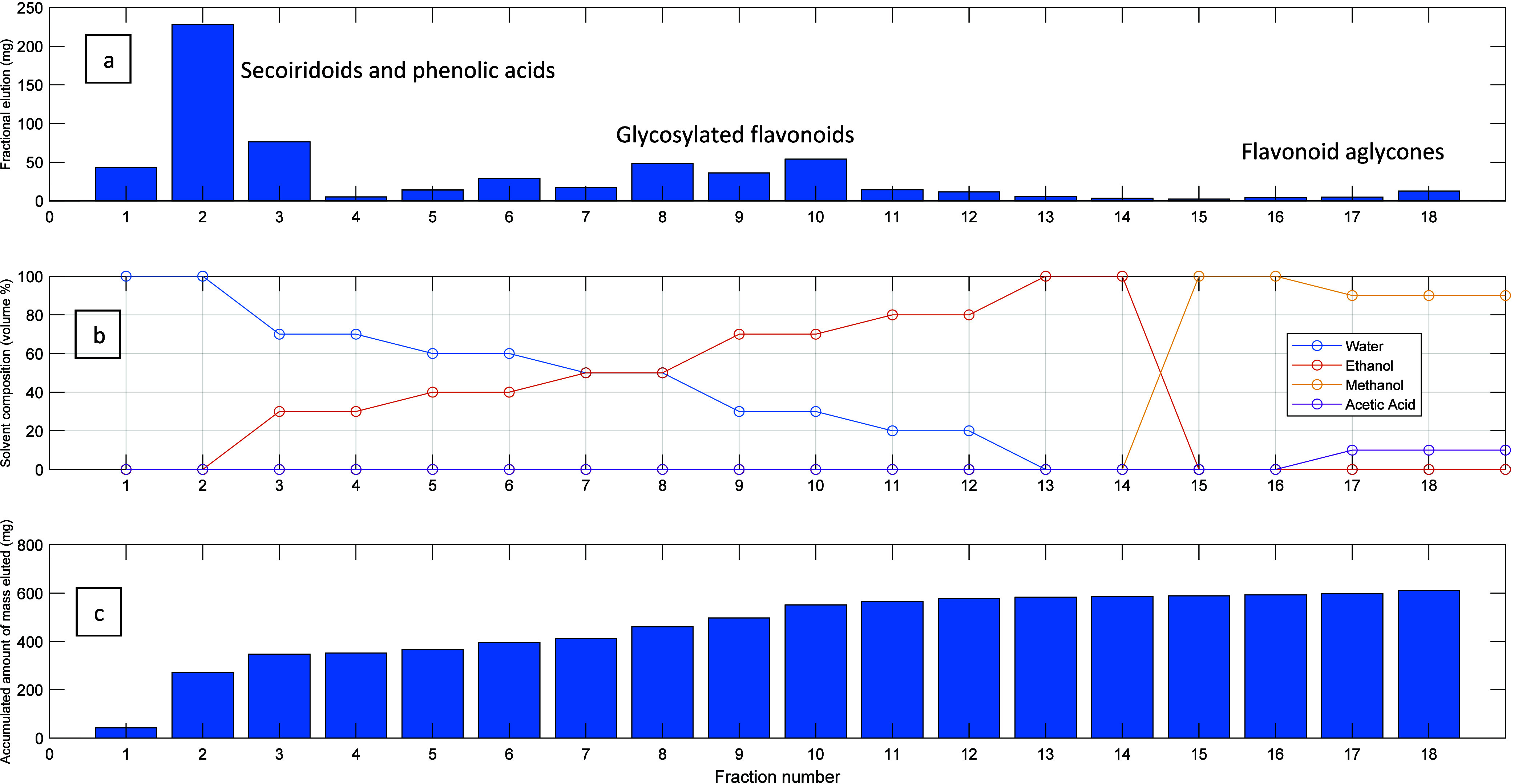
Desorption step for the fractionation of the OPA 20% extract with a quercetin MIP packed in a preparative column. The designed process includes solvent gradient desorption at 45 °C (the extract in EtOH/water at 5 mg/mL was previously loaded to the column at 25 °C). A total of 18 different fractions were produced in this run corresponding to the elution of ca. 664 mg of accumulated mass ((a) stands for the mass of each fraction, (b) for the solvent gradient, and (c) for the accumulated amount produced).

Figures 7 and 8 illustrate the approach here considered for the fractionation of the OPA NATAC 20% extract through a sorption–desorption process with the developed MIP particles packed in a preparative column (25 g of adsorbent). In Figure 7 is presented the UV–vis monitoring of the loading process working with the extract in EtOH/water at 5 mg/mL and at *T* = 25 °C. Figure 8 describes the subsequent desorption step with a solvent gradient at 45 °C. Figure 8(a) stands for the mass of each fraction produced (mg), Figure 8(b) for the solvent gradient considered (composition of the elution solvent in vol %), and Figure 8(c) for the accumulated amount of mass eluted (mg). Globally, 18 different samples were obtained with an accumulated eluted mass of 664 mg. The solvent-gradient process adopted for desorption includes first stages with water (fractions 1 and 2) and afterward the elution with mixed compositions of water/ethanol up to pure ethanol (fractions 13 and 14). At the later stages, the column was cleaned with methanol (fractions 15 and 16) and methanol/acetic acid (fractions 17 and 18).

**Figure 9:**
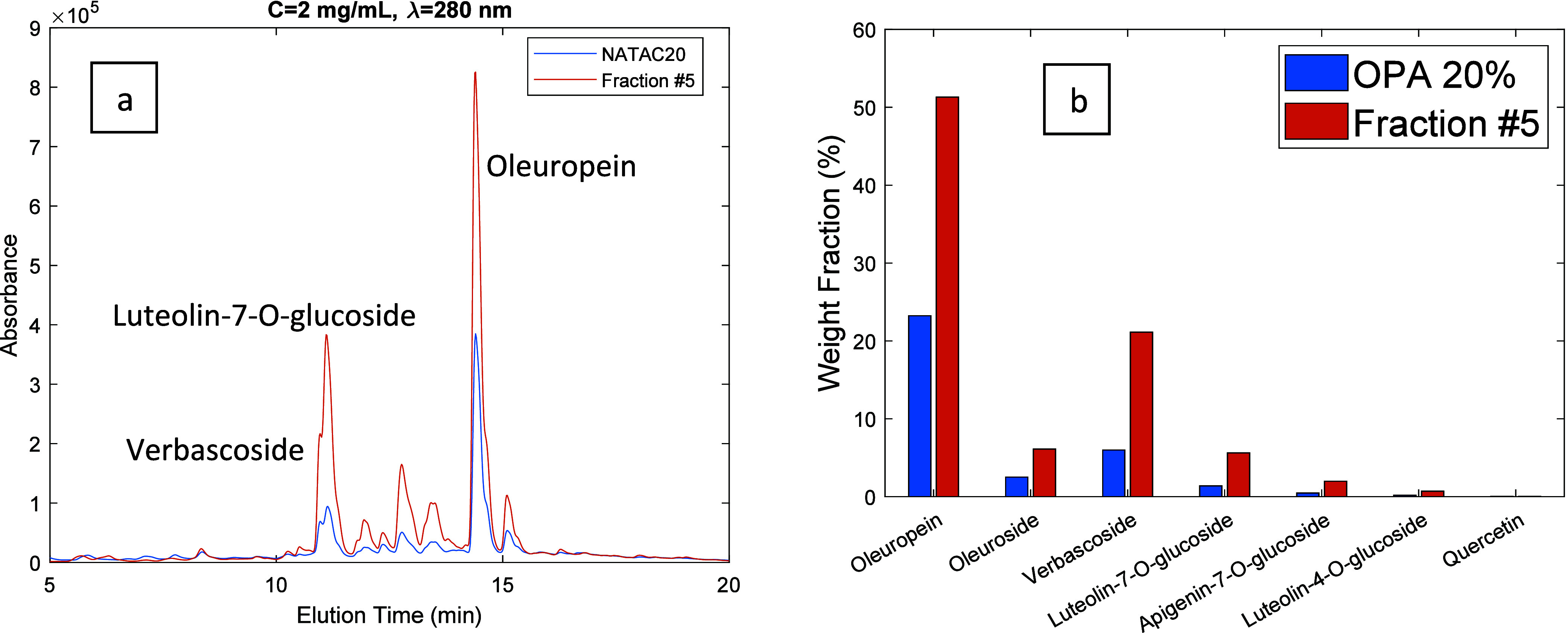
(a) HPLC-DAD chromatograms (λ = 280 nm) for the OPA 20% olive leaf extract (blue line) and for fraction 5 produced through the processing of the extract with the developed MIP. Both products were injected at a concentration of 2 mg/mL. (b) Comparison between the weight fraction (%) of leading phenolic compounds in the OPA20% olive leaf extract (blue bars) and the produced fraction (orange bars).

**Figure 10:**
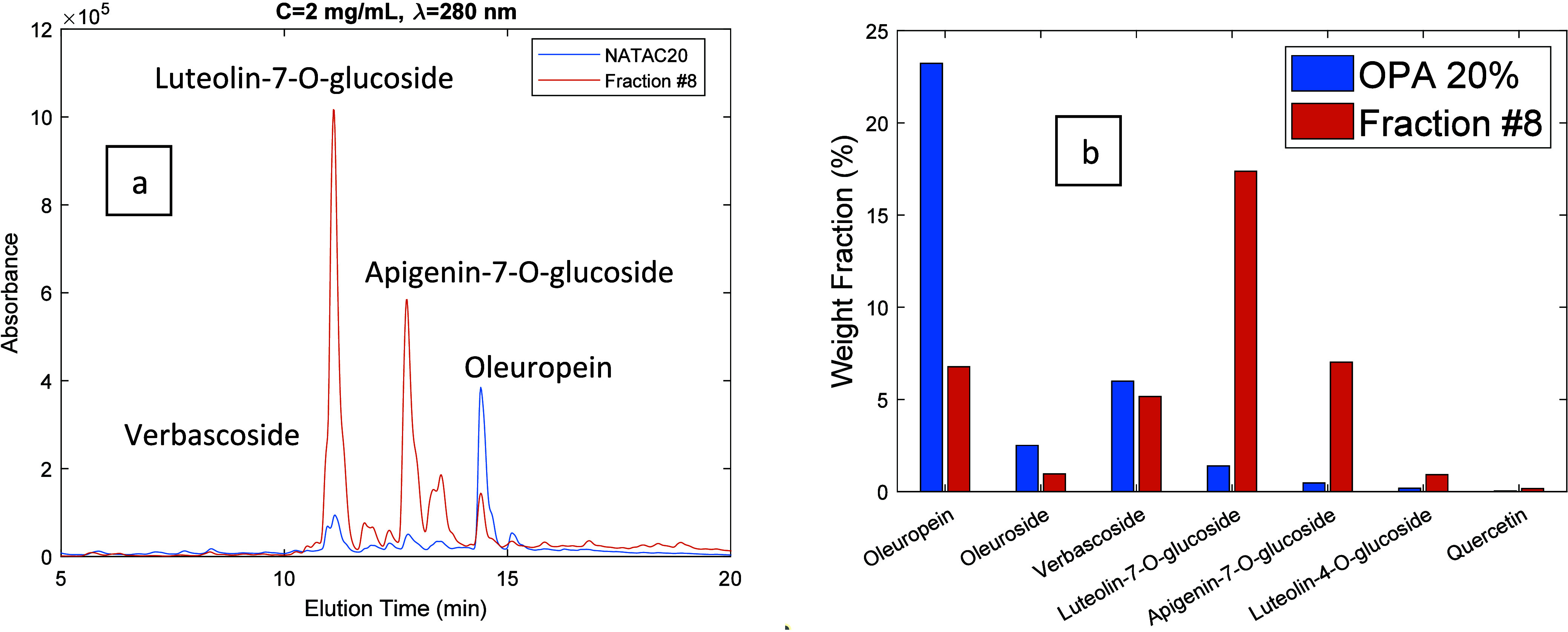
(a) HPLC-DAD chromatograms (λ=280 nm) for the OPA 20% olive leaf extract (blue line) and for fraction 8 produced through the processing of the extract with the developed MIP. Both products were injected at a concentration of 2 mg/mL. (b) Comparison between the weight fraction (%) of leading phenolic compounds in the OPA 20% olive leaf extract (blue bars) and in the produced fraction (orange bars).

**Figure 11:**
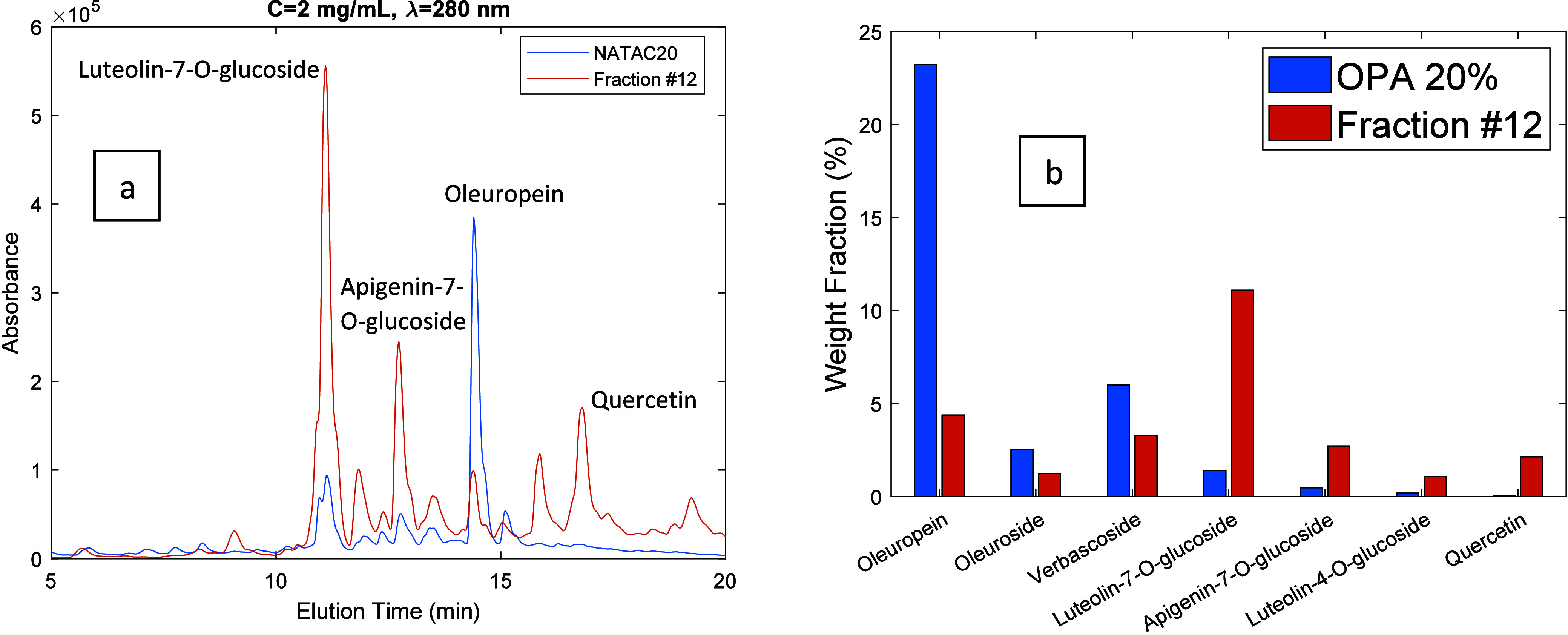
(a) HPLC-DAD chromatograms (λ = 280 nm) for the OPA 20% olive leaf extract (blue line) and for fraction 12 produced through the processing of the extract with the developed MIP. Both products were injected at a concentration of 2 mg/mL. (b) Comparison between the weight fraction (%) of leading phenolic compounds in the OPA 20% olive leaf extract (blue bars) and in the produced fraction (orange bars).

**Figure 12:**
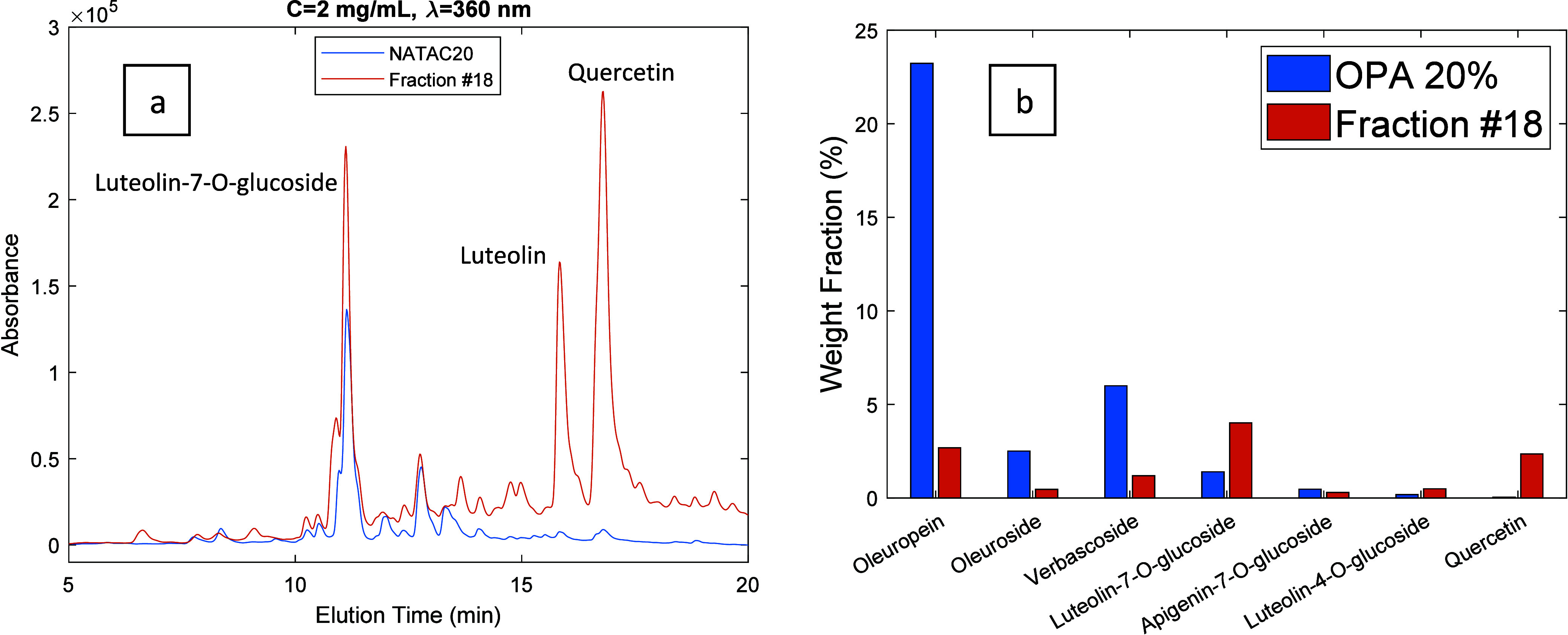
(a) HPLC-DAD chromatograms (λ = 360 nm) for the OPA 20% olive leaf extract (blue line) and for fraction 18 produced through the processing of the extract with the developed MIP. Both products were injected at a concentration of 2 mg/mL. (b) Comparison between the weight fraction (%) of leading phenolic compounds in the OPA 20% olive leaf extract (blue bars) and in the produced fraction (orange bars).

The different fractions collected in the desorption step were dried, and the recovered solid was prepared for HPLC-DAD analysis (all the samples were injected at concentration of 2 mg/mL), aiming at the composition determination and comparison with the original olive leaf extract for fractionation assessment. The achievements of such process are highlighted in Figure 6 and Figures 9–12. Figure 6 demonstrates, through the presented 3D chromatograms, the relevant enrichment obtained for flavonoids in fractions 8, 12, and 18 (see Figure 8 for desorption details). Visual comparison of Figure 6(a) with Figure 6(b–d) clearly elucidates these outcomes. Fraction 8 is extremely rich in luteolin-7-*O*-glucoside when compared with the olive leaf extract OPA 20%, fraction 12 reveals a mixed composition in flavonoid glycosides (luteolin-7-*O*-glucoside and apigenin-7-*O*-glucoside) and aglycone flavonoids (luteolin, quercetin), while fraction 18 is highly enriched with the latter class of compounds.

In Figures 9–12 are presented 2D views for the comparison of the HPLC-DAD chromatogram of the OPA 20% extract and selected fractions obtained through the desorption process. These plots also include the comparison between the weight fraction (%) of leading phenolic compounds in the OPA 20% olive leaf extract and in the produced fractions. Weight fractions for the different compounds were estimated from the HPLC-DAD analysis and the corresponding calibration lines. Note the important enrichment achieved in the different fractions comparatively to the original extract, namely, in fraction 5 (Figure 9) for the secoiridoids oleuropein, and oleuroside and also for verbascoside. For instance, the weight fraction of oleuropein in the OPA 20% extract is estimated to be 23% while in fraction 5 it was measured to be 51% (see bars in Figure 9), corresponding therefore to a calculated enrichment factor of 2.22. Globally, with fractions collected with ethanol content <40% is observed an important enrichment for secoiridoids, phenolic acids, and verbascoside (e.g., enrichment factor up to 5), as shown in Figure 8(a). The reasoning for such an outcome is the lower binding strength of these classes of compounds with the MIP particles, as discussed above.

When the composition of the eluent is in the range 40% < alcohol content < 80%, fractions strongly enriched in glycosylated flavonoids are produced, as shown in Figure 8(a) and highlighted with specific examples in Figure 10 and Figure 11. For instance, enrichment factors of 12.5 and 11.7 were measured for luteolin-7-*O*-glucoside and apigenin-7-*O*-glucoside, respectively. Glycosylated flavonoids strongly bind to the MIP particles compared to secoiridoids and phenolic acids due to the flavonoid core moiety and therefore are eluted later in the desorption process.

Flavonoid aglycones such as luteolin and quercetin were enriched in fractions with alcohol content >80% as noted in Figure 8(a) and highlighted with specific examples in Figure 12 (see also Figures 6(c,d)). Notably, enrichment factors >20 for luteolin and quercetin were estimated in these later desorbed fractions. This is clearly a consequence of the very high binding strength with the MIP particles observed for flavonoid aglycones, as demonstrated above, considering the competitive adsorption studies.

Estimated productivity in terms of the mass of phenolic compounds processed per mass of adsorbent indicates a global threshold of 27 mg/g and a value close to 1.5 mg/g if luteolin-7-*O*-glucoside is taken as reference. These values are much higher than those observed with hybrid cellulose-synthetic MIPs recently addressed to target the same extract (e.g., up to 0.2 mg/g)^12^ but at the expenses of using a totally synthetic particles.

Improved separation and purification of the olive leaf contained phenolic compounds is possible, if required by the final application of the products, namely, for food, feed, pharmaceuticals, or cosmetics industries that often consider very different purity grades. The repeated processing of the fractions illustrated here with the same MIP adsorbent is a possibility for enhancement of the purity of the target compounds. Also, with the same adsorbent, the redesign of the extract sorption/desorption conditions (see Figures 7 and 8) can be considered to tailor the compositions of the produced fractions. This approach can also be considered in complement with other purification techniques (e.g., membrane nanofiltration) and in a train with other kinds of tailored adsorbents specifically designed to target compounds in olive leaf.^13,14^

The reproducibility of the sorption/desorption approach here proposed is a key aspect if the industrial application is aimed. Therefore, repeated experiments were performed in this context and the correspondent results are presented in the Supporting Information, namely, in Figures S11 and S12. These results, in combination with those presented in Figure 8, demonstrate the reproducibility of the fractionation method developed, concerning not only the mass of extract processed (626 ± 38 mg) but also the design of the composition of products. Indeed, the production of phenolic acids, secoiridoids, glycosylated flavonoids, and the related aglycones was observed in the repeated experiments. A total of 148 different fractions were produced and analyzed in the repeated experiments reported in Figure 8 and Figures S11 and S12. A total of eight independent experiments similar to those depicted in Figure 8 and Figures S11 and S12 were performed with real olive leaf extracts, demonstrating the good reproducibility of the process and reusability of the developed MIP particles (see a comprehensive discussion on the long-term stability and reusability of MIPs in ref (40)).

Overall, the findings reported here demonstrate that the tailored materials and sorption/desorption conditions we developed can play an important role for process intensification in the framework of circular bioeconomy aiming at the valorization of olive leaf. This approach is also potentially extensible to the targeting of other kinds of agricultural residues containing phenolic compounds.

## 4 Conclusion

A molecularly imprinted polymer network for quercetin functionalized with 4-vinylpiridine moieties was synthesized through inverse suspension polymerization to be used as an adsorbent for the fractionation of phenolic compounds in olive leaf. The competitive adsorption of phenolic acids, secoiridoids, and flavonoids in the developed material was studied considering the standard molecules vanillic acid, oleuropein, and quercetin. The measured adsorption isotherms highlight a much stronger binding capacity of the quercetin-MIP particles toward quercetin as compared with vanillic acid and oleuropein. The acquired data were used to design and scale-up sorption/desorption processes aiming at the fractionation of olive leaf extracts. Within this purpose, 25 g of the synthesized MIP adsorbent were packed in a preparative column.

It was demonstrated that a simple adsorption process is feasible when working with an industrial olive leaf extract at high extract concentration (e.g., 5–10 mg/mL) due to the strong binding capacity of the MIP for flavonoids, even when using aqueous mixtures with a large alcoholic content (e.g., ethanol volumetric fraction higher than 50%). Solvent-gradient and temperature-swing drive desorption runs were performed and lead to the production of a sequence of fractions with very different compositions comparatively to the initial extract. Non-flavonoid compounds, such as oleuropein and verbascoside, were enriched in fractions with low alcoholic content while glycosylated flavonoids were strongly enriched in fractions with 40% < alcohol content < 80%. (e.g., enrichment factors of 12.5 and 11.7 were measured for luteolin-7-*O*-glucoside and apigenin-7-*O*-glucoside, respectively). Furthermore, flavonoid aglycones such as luteolin and quercetin were enriched in fractions with alcohol content >80% (e.g., enrichment factors >20 were estimated for luteolin and quercetin).

The findings reported here demonstrate the usefulness of the developed materials and sorption/desorption conditions for process intensification in the framework of circular bioeconomy, namely, to get high-added-value compounds from olive leaf or other kinds of agricultural residues.

## Supporting Information Available

Figures S1–S10, data for the dynamics of competitive adsorption and desorption of vanillic acid, oleuropein, and quercetin in molecularly imprinted polymers; Figures S11 and S12, results for the fractionation of olive leaf extracts with molecularly imprinted polymers; E1, calculation example for the quantification of the uncertainties associated to the MIP adsorbed amounts (PDF)
