# Supplementary material for: Competitive Adsorption of Phenolic Acids, Secoiridoids, and Flavonoids in Quercetin Molecularly Imprinted Polymers and Application for Fractionation of Olive Leaf Extracts
Source: J Chem Eng Data. 2024 Feb 28;69(10):3629–44. doi: 10.1021/acs.jced.3c00543 (PMC11472317; doi:10.1021/acs.jced.3c00543)
Supplement: Supplementary file 1 — je3c00543_si_001.pdf [file je3c00543_si_001.pdf]

Supporting Information

Competitive Adsorption of Phenolic Acids, Secoiridoids and Flavonoids in Quercetin Molecularly Imprinted Polymers and Application for Fractionation of Olive Leaf Extracts

Ayssata Almeida[1], Cláudia Martins, Rolando C. S. Dias[1*], Mário Rui P. F. N. Costa[2]

[1]Centro de Investigação de Montanha (CIMO), Instituto Politécnico de Bragança,

Campus de Santa Apolónia, 5300-253 Bragança, Portugal

[2]LSRE, Faculdade de Engenharia da Universidade do Porto, Rua Roberto Frias s/n,

4200-465 Porto, Portugal

* Correspondence to Rolando C. S. Dias, rdias@ipb.pt

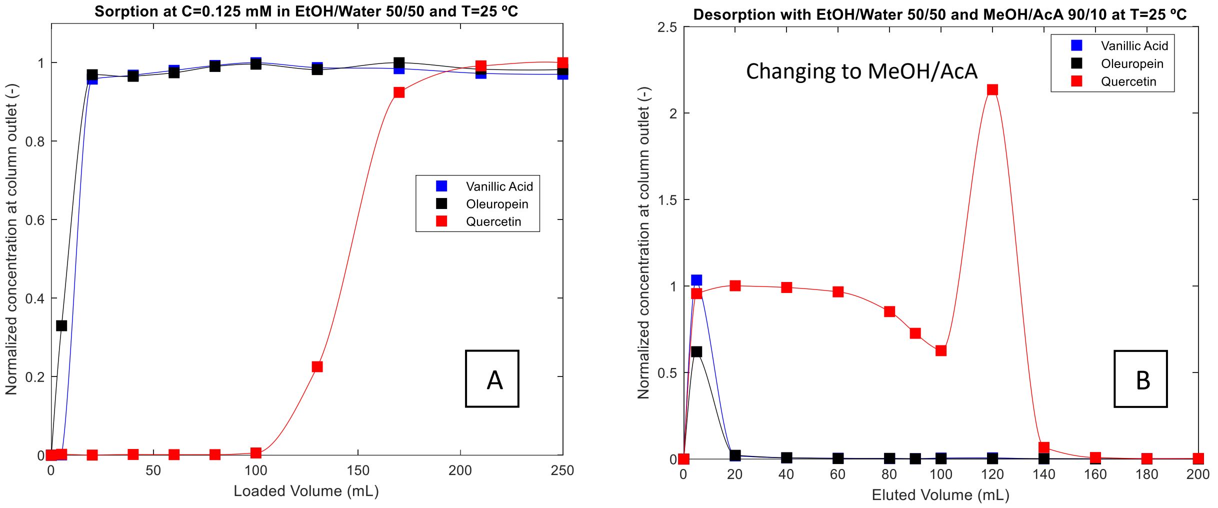

Figure S1: A: Experimental breakthrough curve for the competitive adsorption of a solution in ethanol/water 50/50 of vanillic acid, oleuropein and quercetin, at a concentration C=0.125 mM for each compound, in a column (L=50 mm, D=4.6 mm) containing 290 mg of MIP particles: Running conditions: Q=1 mL/min, T=25 °C (Run #1). B: Experimental desorption profile for the same experiment including a first stage with ethanol/water 50/50 as eluent and after methanol/acetic acid 90/10, both steps at 25 ºC. Lines in the plots were included only to guide the eyes.

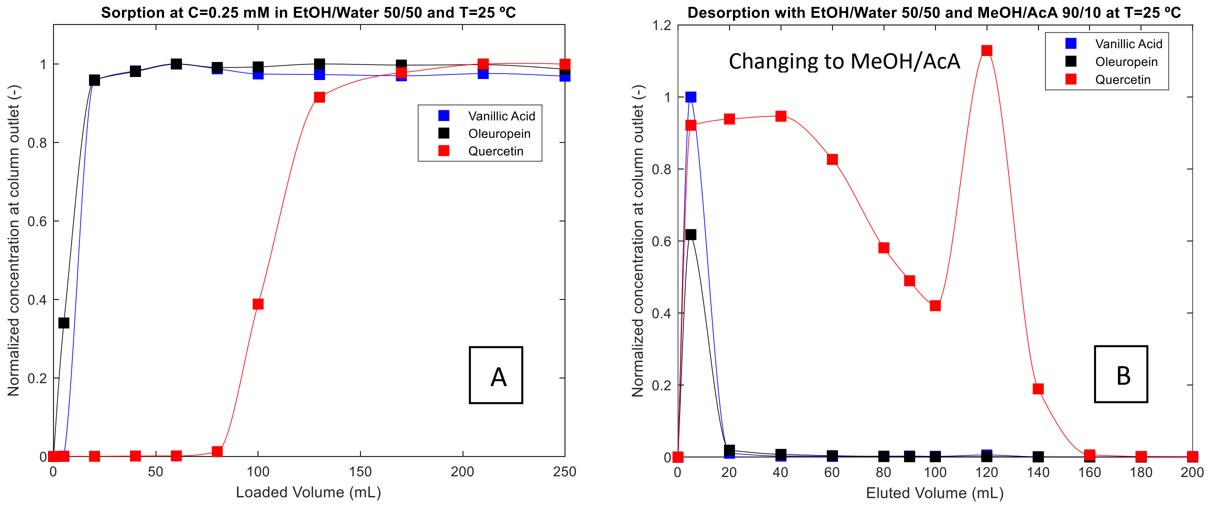

Figure S2: A: Experimental breakthrough curve for the competitive adsorption of a solution in ethanol/water 50/50 of vanillic acid, oleuropein and quercetin, at a concentration C=0.25 mM for each compound, in a column (L=50 mm, D=4.6 mm) containing 290 mg of MIP particles: Running conditions: Q=1 mL/min, T=25 °C (Run #2). B: Experimental desorption profile for the same experiment including a first stage with ethanol/water 50/50 as eluent and after methanol/acetic acid 90/10, both steps at 25 ºC. Lines in the plots were included only to guide the eyes.

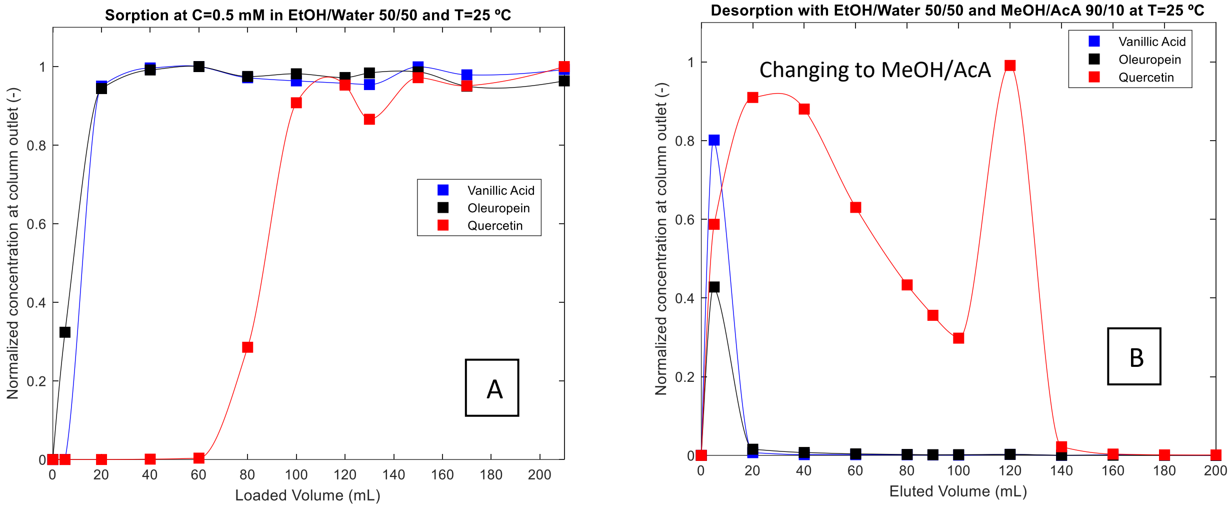

Figure S3: A: Experimental breakthrough curve for the competitive adsorption of a solution in ethanol/water 50/50 of vanillic acid, oleuropein and quercetin, at a concentration C=0.5 mM for each compound, in a column (*L*=50 mm, *D*=4.6 mm) containing 290 mg of MIP particles: Running conditions: *Q*=1 mL/min, *T*=25 °C (Run #3). B: Experimental desorption profile for the same experiment including a first stage with ethanol/water 50/50 as eluent and after methanol/acetic acid 90/10, both steps at 25 ºC. Lines in the plots were included only to guide the eyes.

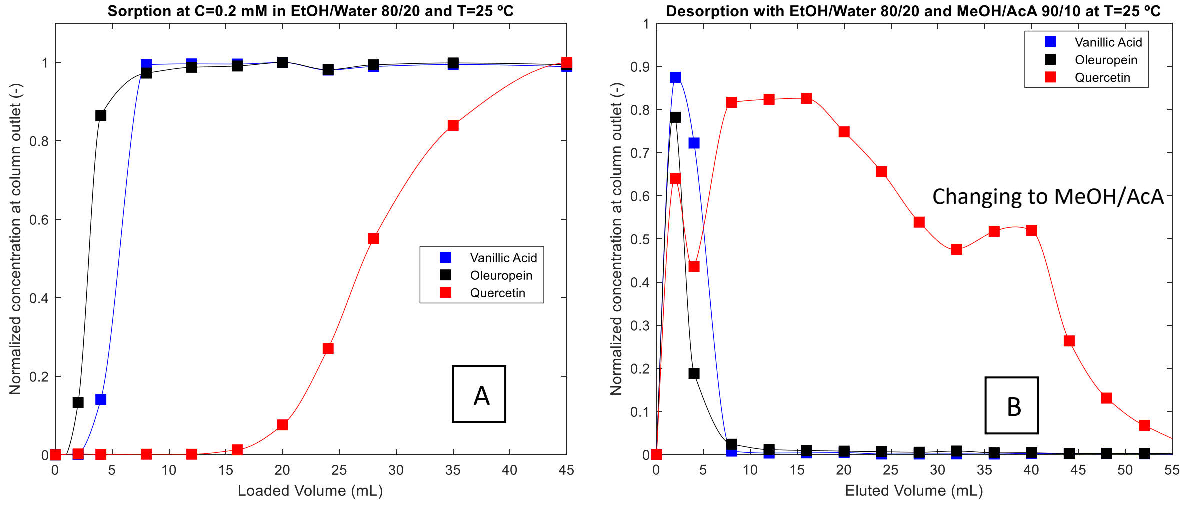

Figure S4: A: Experimental breakthrough curve for the competitive adsorption of a solution in ethanol/water 80/20 of vanillic acid, oleuropein and quercetin, at a concentration C=0.2 mM for each compound, in a column (*L*=50 mm, *D*=4.6 mm) containing 290 mg of MIP particles: Running conditions: *Q*=1 mL/min, *T*=25 °C (Run #4). B: Experimental desorption profile for the same experiment including a first stage with ethanol/water 80/20 as eluent and after methanol/acetic acid 90/10, both steps at 25 ºC. Lines in the plots were included only to guide the eyes.

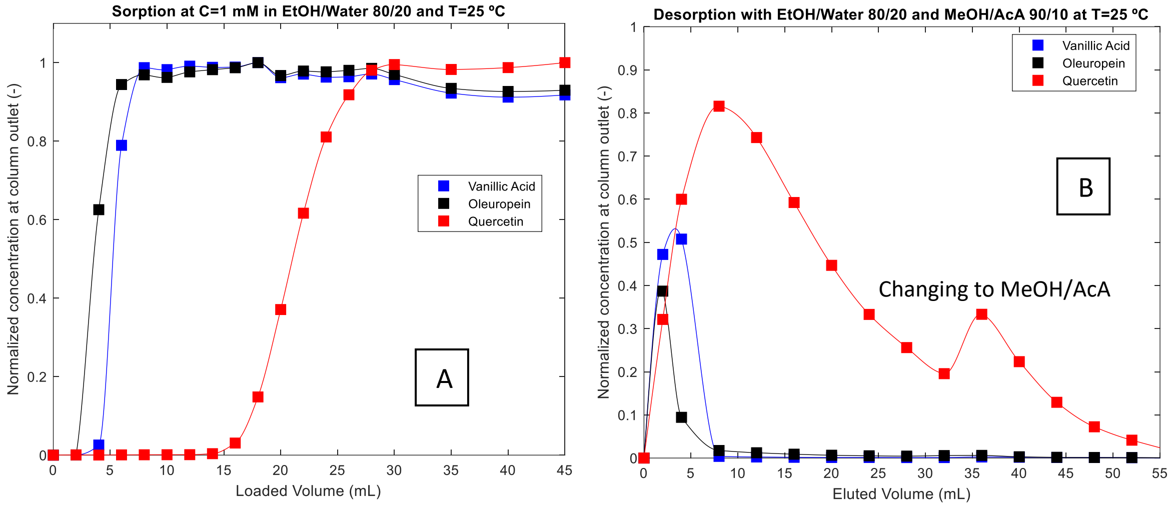

Figure S5: A: Experimental breakthrough curve for the competitive adsorption of a solution in ethanol/water 80/20 of vanillic acid, oleuropein and quercetin, at a concentration C=1 mM for each compound, in a column (L=50 mm, D=4.6 mm) containing 290 mg of MIP particles: Running conditions: Q=1 mL/min, T=25 °C (Run #5). B: Experimental desorption profile for the same experiment including a first stage with ethanol/water 80/20 as eluent and after methanol/acetic acid 90/10, both steps at 25 ºC. Lines in the plots were included only to guide the eyes.

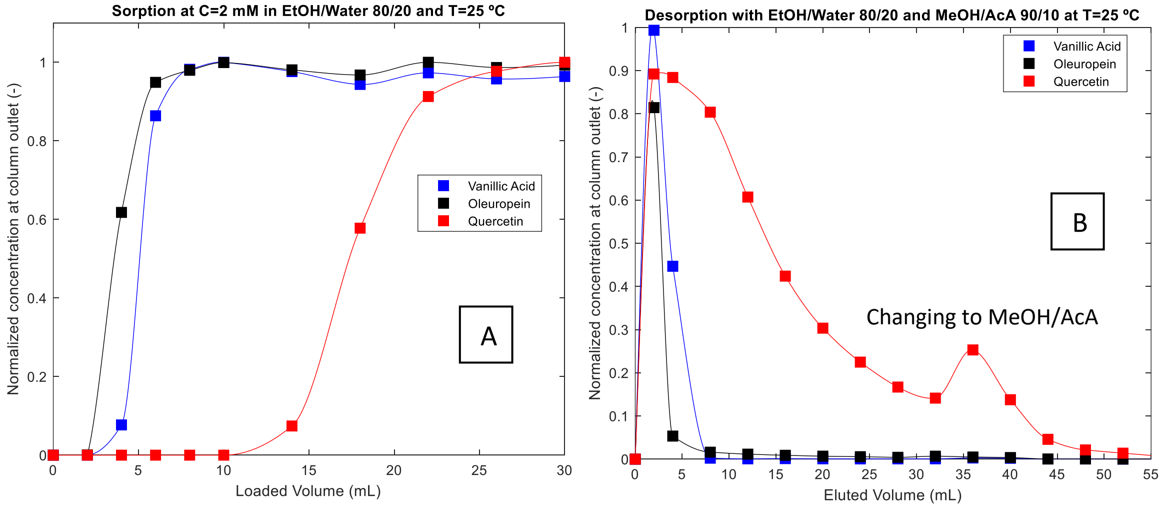

Figure S6: A: Experimental breakthrough curve for the competitive adsorption of a solution in ethanol/water 80/20 of vanillic acid, oleuropein and quercetin, at a concentration C=2 mM for each compound, in a column (L=50 mm, D=4.6 mm) containing 290 mg of MIP particles: Running conditions: Q=1 mL/min, T=25 °C (Run #6). B: Experimental desorption profile for the same experiment including a first stage with ethanol/water 80/20 as eluent and after methanol/acetic acid 90/10, both steps at 25 ºC. Lines in the plots were included only to guide the eyes.

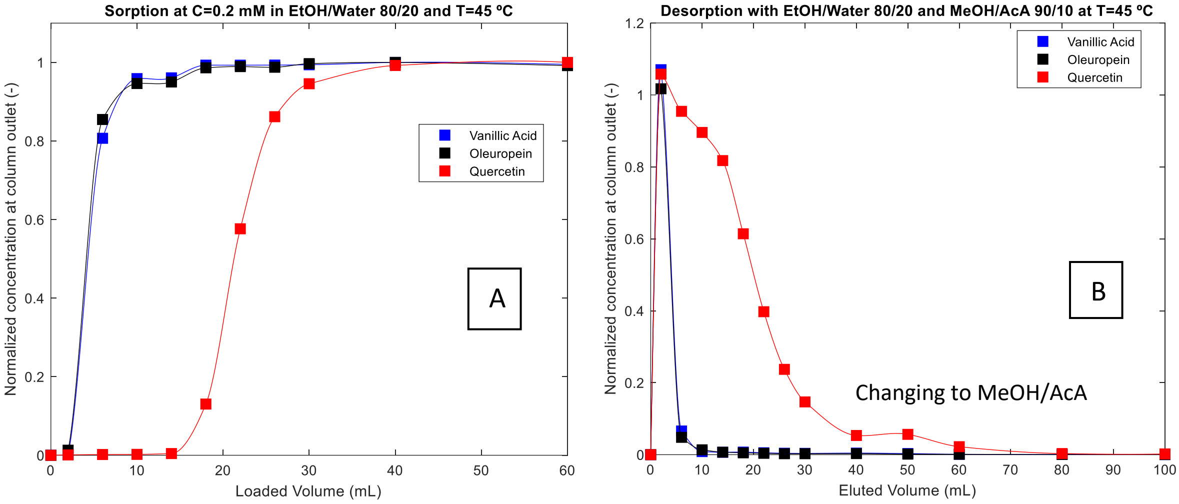

Figure S7: A: Experimental breakthrough curve for the competitive adsorption of a solution in ethanol/water 80/20 of vanillic acid, oleuropein and quercetin, at a concentration C=0.2 mM for each compound, in a column (*L*=50 mm, *D*=4.6 mm) containing 290 mg of MIP particles: Running conditions: *Q*=1 mL/min, *T*=45 °C (Run #7). B: Experimental desorption profile for the same experiment including a first stage with ethanol/water 80/20 as eluent and after methanol/acetic acid 90/10, both steps at 45 ºC. Lines in the plots were included only to guide the eyes.

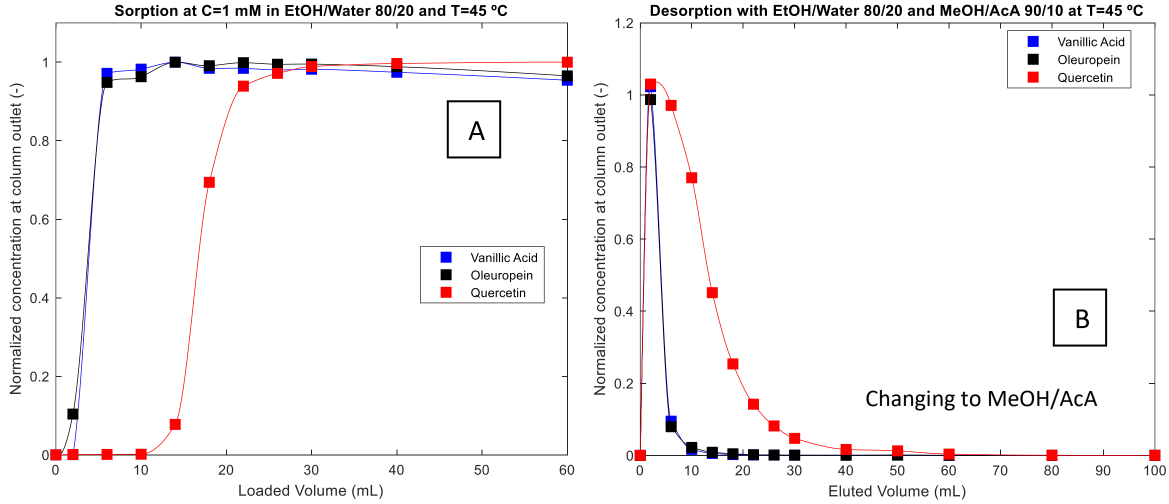

Figure S8: A: Experimental breakthrough curve for the competitive adsorption of a solution in ethanol/water 80/20 of vanillic acid, oleuropein and quercetin, at a concentration C=1 mM for each compound, in a column (*L*=50 mm, *D*=4.6 mm) containing 290 mg of MIP particles: Running conditions: *Q*=1 mL/min, *T*=45 °C (Run #8). B: Experimental desorption profile for the same experiment including a first stage with ethanol/water 80/20 as eluent and after methanol/acetic acid 90/10, both steps at 45 ºC. Lines in the plots were included only to guide the eyes.

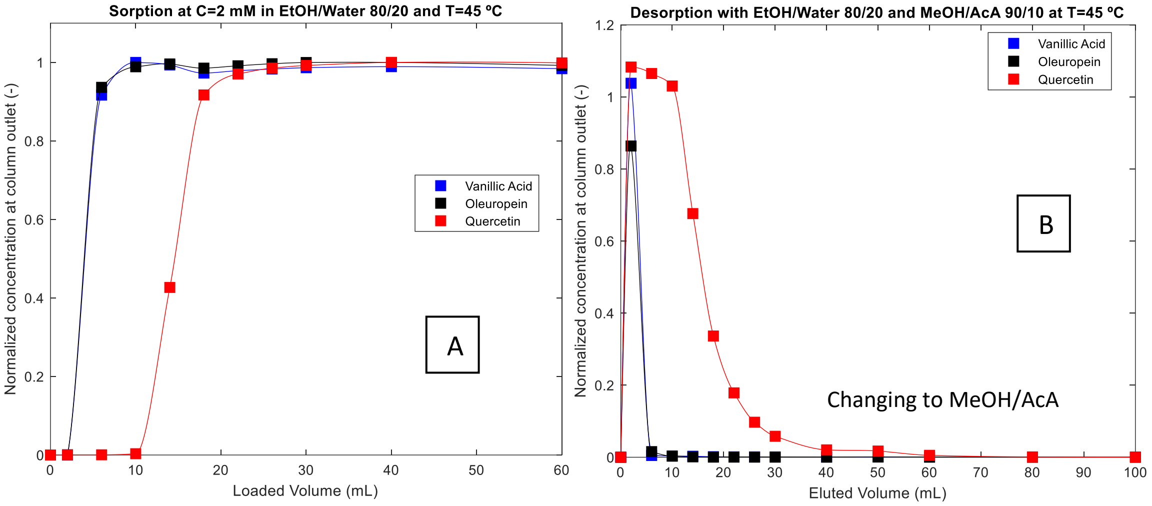

Figure S9: A: Experimental breakthrough curve for the competitive adsorption of a solution in ethanol/water 80/20 of vanillic acid, oleuropein and quercetin, at a concentration C=2 mM for each compound, in a column (L=50 mm, D=4.6 mm) containing 290 mg of MIP particles: Running conditions: Q=1 mL/min, T=45 °C (Run #9). B: Experimental desorption profile for the same experiment including a first stage with ethanol/water 80/20 as eluent and after methanol/acetic acid 90/10, both steps at 45 ºC. Lines in the plots were included only to guide the eyes.

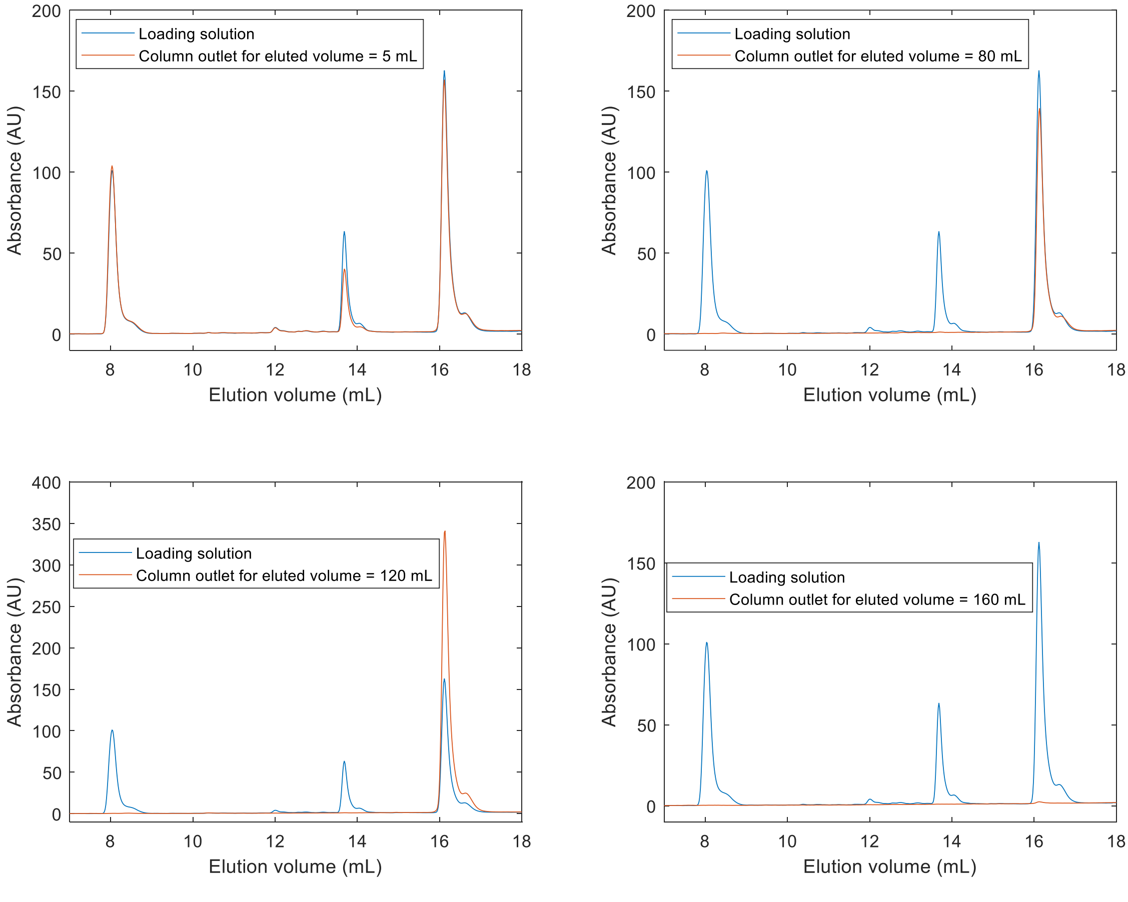

Figure S10: HPLC analysis for samples collected at MIP column outlet during the desorption of the MIP column previously saturated with a solution containing vanillic acid, oleuropein and quercetin according to conditions of Run #1.

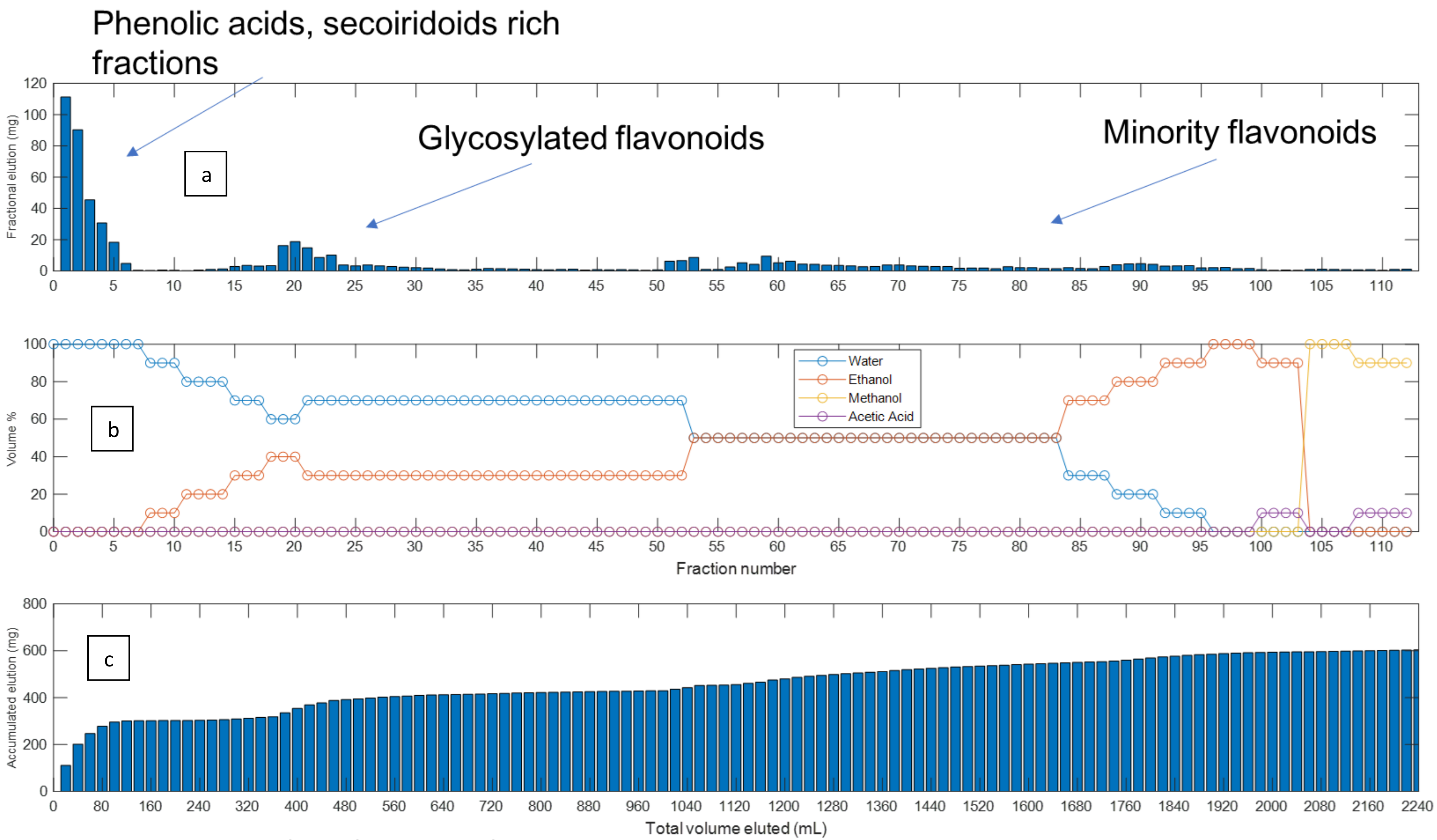

Figure S11. Desorption step for the fractionation of the OPA 20% extract with a quercetin MIP packed in a preparative column. The designed process includes a solvent gradient desorption at 45 °C (the extract in EtOH/water at 5 mg/mL was previously loaded to the column at 25 °C). A total of 112 different fractions were produced in this run corresponding to the elution of ca. 604 mg of accumulated mass ((a) stands for the mass of each fraction, (b) for the solvent gradient and (c) for the accumulated amount produced).

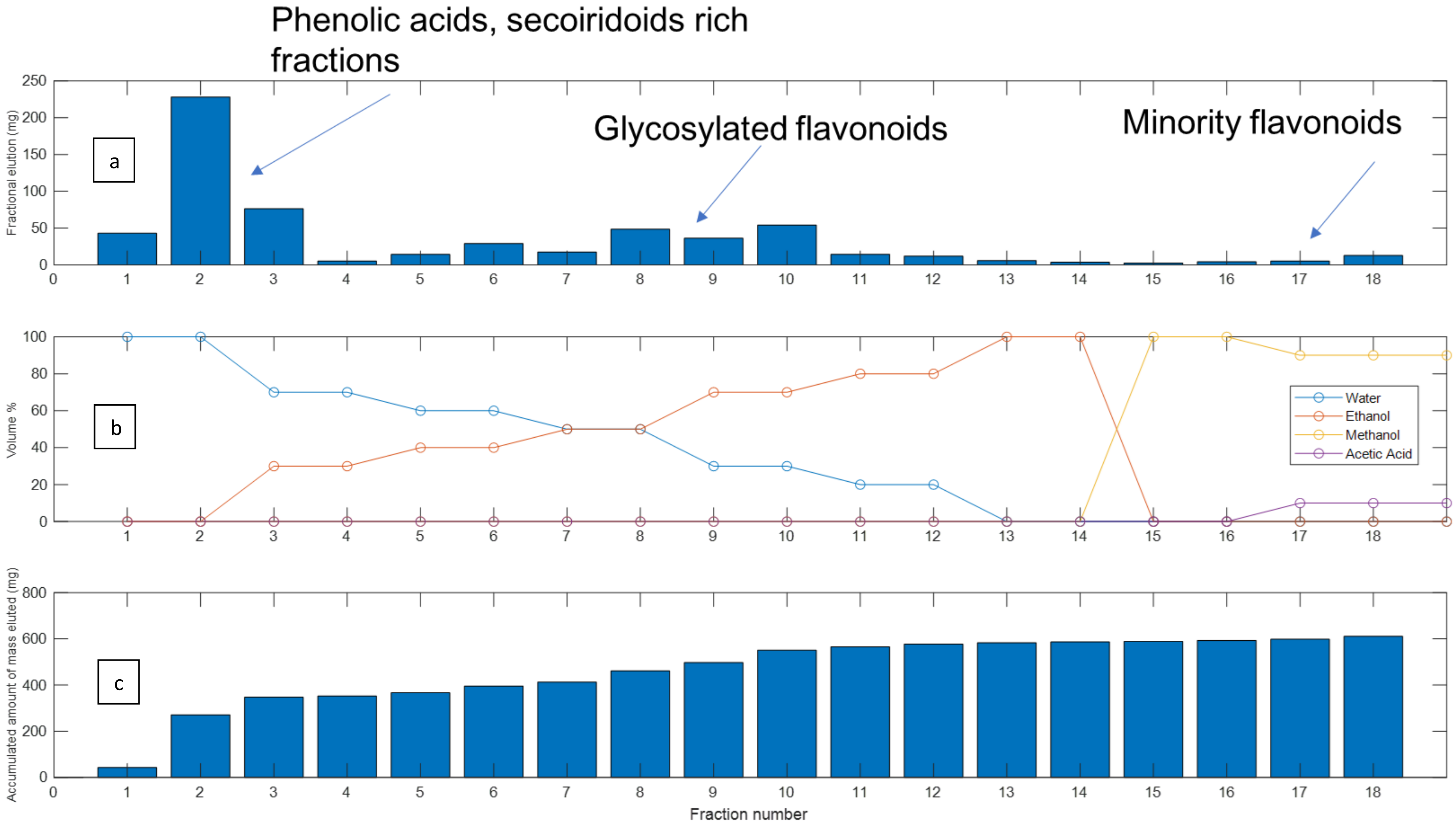

Figure S12. Desorption step for the fractionation of the OPA 20% extract with a quercetin MIP packed in a preparative column. The designed process includes a solvent gradient desorption at 45 °C (the extract in EtOH/water at 5 mg/mL was previously loaded to the column at 25 °C). A total of 18 different fractions were produced in this run corresponding to the elution of ca. 611 mg of accumulated mass ((a) stands for the mass of each fraction, (b) for the solvent gradient and (c) for the accumulated amount produced).

E1

Calculation example for the quantification of the uncertainties associated to the MIP adsorbed amounts considering the runs with standard polyphenols. Run#1 with quercetin molecule is here considered for illustration purposes.

Solute concentration (C)	0.125	mM					
Volume percolated in adsorption (Va)	250	mL					
Volume percolated in adsorption (Vd)	200	mL					
Adsorbent mass (m)	295	mg					
HPLC Calibration constant for Area=k*C	16875	AU*time/mM					
					Adsorbed amount		
HPLC-DAD analysis of global liquid solution after percolation (Aads)	825	AU*time			q=Va*1e-3*(C-Aads/k)/m*1e6	64.5	umol/g
HPLC-DAD of desorbed fractions (Ades)	1590	AU*time			q=Vd*1e-3*(Ades/k)/m*1e6	63.9	umol/g
Area sorption makima/trapz (A1)	110	Total area (AT)	250		q=Va*(1-A1/AT)*C*1e-3/m*1e6	59.3	umol/g
Area desorption makima/trapz (A2)	135				q=C*1e-3*A2/m*1e6	57.2	umol/g
					average	61.2	umol/g
					SD	3.5	umol/g
					Uncertainty (SD/sqrt(4))	1.8	umol/g
